# Asynchronous and Coherent Dynamics in Balanced Excitatory-Inhibitory Spiking Networks

**DOI:** 10.3389/fnsys.2021.752261

**Published:** 2021-12-10

**Authors:** Hongjie Bi, Matteo di Volo, Alessandro Torcini

**Affiliations:** ^1^CY Cergy Paris Université, Laboratoire de Physique Théorique et Modélisation, CNRS, UMR 8089, Cergy-Pontoise, France; ^2^Neural Coding and Brain Computing Unit, Okinawa Institute of Science and Technology, Okinawa, Japan; ^3^CNR-Consiglio Nazionale delle Ricerche, Istituto dei Sistemi Complessi, Sesto Fiorentino, Italy

**Keywords:** balanced spiking neural populations, sparse inhibitory-excitatory networks, asynchronous dynamics, collective oscillations, neural mass model, quadratic integrate and fire neuron, structural heterogeneity, coherent chaos

## Abstract

Dynamic excitatory-inhibitory (E-I) balance is a paradigmatic mechanism invoked to explain the irregular low firing activity observed in the cortex. However, we will show that the E-I balance can be at the origin of other regimes observable in the brain. The analysis is performed by combining extensive simulations of sparse E-I networks composed of *N* spiking neurons with analytical investigations of low dimensional neural mass models. The bifurcation diagrams, derived for the neural mass model, allow us to classify the possible asynchronous and coherent behaviors emerging in balanced E-I networks with structural heterogeneity for any finite in-degree *K*. Analytic mean-field (MF) results show that both supra and sub-threshold balanced asynchronous regimes are observable in our system in the limit *N* >> *K* >> 1. Due to the heterogeneity, the asynchronous states are characterized at the microscopic level by the splitting of the neurons in to three groups: silent, fluctuation, and mean driven. These features are consistent with experimental observations reported for heterogeneous neural circuits. The coherent rhythms observed in our system can range from periodic and quasi-periodic collective oscillations (COs) to coherent chaos. These rhythms are characterized by regular or irregular temporal fluctuations joined to spatial coherence somehow similar to coherent fluctuations observed in the cortex over multiple spatial scales. The COs can emerge due to two different mechanisms. A first mechanism analogous to the pyramidal-interneuron gamma (PING), usually invoked for the emergence of γ-oscillations. The second mechanism is intimately related to the presence of current fluctuations, which sustain COs characterized by an essentially simultaneous bursting of the two populations. We observe period-doubling cascades involving the PING-like COs finally leading to the appearance of coherent chaos. Fluctuation driven COs are usually observable in our system as quasi-periodic collective motions characterized by two incommensurate frequencies. However, for sufficiently strong current fluctuations these collective rhythms can lock. This represents a novel mechanism of frequency locking in neural populations promoted by intrinsic fluctuations. COs are observable for any finite in-degree *K*, however, their existence in the limit *N* >> *K* >> 1 appears as uncertain.

## 1. Introduction

Cortical neurons are subject to a continuous bombardment from thousands of presynaptic neurons, mostly pyramidal ones, evoking postsynaptic potentials of sub-millivolt or millivolt amplitudes (Destexhe and Paré, 1999; Bruno and Sakmann, 2006; Lefort et al., 2009). This stimulation would induce an almost constant depolarization of the neurons leading to a regular firing, However, cortical neurons fire quite irregularly and with low firing rates (Softky and Koch, 1993). This apparent paradox can be solved by introducing the concept of a balanced network, where excitatory and inhibitory synaptic currents are approximately balanced and the neurons are kept near their firing threshold crossing it at random times (Shadlen and Newsome, 1994, 1998). However, the balance should naturally emerge in the network without fine-tuning of the parameters and the highly irregular firing observed *in vivo* should be maintained also for a large number of connections (in-degree) *K* >> 1. This is possible by considering a sparse excitatory-inhibitory (E-I) neural network composed of *N* neurons and characterized by an average in-degree *K* << *N* and by synaptic couplings scaling as $1/\sqrt{K}$ (van Vreeswijk and Sompolinsky, 1996). This scaling as well as many other key predictions of the theory developed in (van Vreeswijk and Sompolinsky, 1996) have been recently confirmed by experiments on a neural culture (Barral and Reyes, 2016). Furthermore, Barral and Reyes (2016) have shown that the major predictions of the seminal theory (van Vreeswijk and Sompolinsky, 1996) also hold under conditions far from the asymptotic limits where *K* and *N* are large.

The dynamics usually observable in balanced neural networks is asynchronous and characterized by irregular neural firing joined to stationary firing rates (van Vreeswijk and Sompolinsky, 1996; Monteforte and Wolf, 2010; Renart et al., 2010; Litwin-Kumar and Doiron, 2012; Ullner et al., 2020). However, other asynchronous regimes characterized by sub-Poissonian and super-Poissonian statistics have been reported in balanced homogenous and heterogeneous networks (Lerchner et al., 2006; Ullner et al., 2020). Furthermore, regular and irregular collective oscillations (COs) have been shown to emerge in balanced networks composed of rate models (van Vreeswijk and Sompolinsky, 1996) and of spiking neurons (Brunel, 2000; Ostojic, 2014; di Volo and Torcini, 2018; Ullner et al., 2018; Bi et al., 2020). The balanced asynchronous irregular state has been experimentally observed both *in vivo* and *in vitro* (Shu et al., 2003; Haider et al., 2006) and dynamic balance of excitation and inhibition is observable in the neocortex across all states of the wake-sleep cycle, in both human and monkey (Dehghani et al., 2016). However, this is not the unique balanced state observable in neural systems. In particular, balancing of excitation and inhibition appears to be crucial for the emergence of cortical oscillations (Okun and Lampl, 2008; Isaacson and Scanziani, 2011; Le Van Quyen et al., 2016) as well as for the instantaneous modulation of the frequency of gamma oscillations in the hippocampus (Atallah and Scanziani, 2009). Moreover, balancing of excitation and inhibition is essential for the generation of respiratory rhythms in the brainstem (Ramirez and Baertsch, 2018) and the rhythmic activity of irregular firing motoneurons in the spinal cord of the turtle (Berg et al., 2007, 2019).

In this work, we characterize in detail the asynchronous regimes and the emergence of COs (population rhythms) in E-I balanced networks with structural heterogeneity. In particular, we consider sparse random networks of quadratic integrate-and-fire (QIF) neurons (Ermentrout and Kopell, 1986) pulse coupled *via* instantaneous post synaptic potentials. We compare numerical findings with analytical results obtained in the mean-field (MF) limit by employing an effective low-dimensional neural mass model recently developed for sparse QIF networks (Montbrió et al., 2015; di Volo and Torcini, 2018; Bi et al., 2020).

In the asynchronous regime, our analytical MF predictions are able to reproduce the mean membrane potentials and the population firing rates of the structurally heterogeneous network for any finite *K* value. Furthermore, in the limit *N* >> *K* >> 1, we analytically derive the asymptotic MF values of the population firing rates and the effective input currents. This analysis shows that the system always achieves balanced dynamics, whose supra or sub-threshold nature is determined by the model parameters. Detailed numerical investigations of the microscopic dynamics allow identifying three different groups of neurons, whose activity is essentially controlled by their in-degrees and by the effective input currents.

In the balanced network, we have identified three types of COs depending on the corresponding solution displayed by the neural mass model. The first type, termed O_*P*_ emerges in the MF *via* a Hopf bifurcation (HB) from a stable focus solution. These COs gives rise to collective chaos *via* a period-doubling sequence of bifurcations. Another type of CO, already reported for pure inhibitory networks (di Volo and Torcini, 2018), denoted as O_*F*_ corresponds in the MF to a stable focus characterized by relaxation oscillations toward the fixed point which in the sparse network become noise sustained oscillations due to fluctuations in the input currents. The last type of COs identified in the finite network are named O_*S*_ and characterized by abnormally synchronized dynamics among the neurons, and the high level of synchronization prevents their representation in the MF formulation (Montbrió et al., 2015).

O_*P*_ and O_*S*_ emerge as sustained oscillations in the network *via* a mechanism resembling that invoked for pyramidal-interneuron gamma (PING) rhythms (Whittington et al., 2011) despite the frequency of these oscillations is not restricted to the γ band. Excitatory neurons start to fire followed by the inhibitory ones and the peak of activity of the excitatory population precedes that of the inhibitory one of a time delay Δ*t*. Furthermore, Δ*t* tends to vanish when the amplitude of the current fluctuations in the network increases. Indeed, for O_*F*_ oscillations, which cannot emerge in absence of current fluctuations, no delay has been observed between the activation of excitatory and inhibitory populations. The last important question that we tried to address in our study was whether the COs, observable for any finite *K*, are still present in the limit *N* >> *K* >> 1.

The study is organized as follows. Section 2 is devoted to the introduction of the network model and of the corresponding effective neural mass model, as well as of the microscopic and macroscopic indicators employed to characterize the neural dynamics. In the same section, the stationary solutions for the balanced neural mass model are analytically obtained as finite in-degree expansion and their range of stability is determined. The macroscopic dynamical regimes emerging in our network are analyzed in section 3. In particular, we report bifurcation phase diagrams obtained from the neural mass model displaying the possible dynamical states and network simulations. The focus of this section is on the analysis of the asynchronous balanced state for structurally heterogeneous networks and the emergence of the different types of COs observable at finite in-degrees. A discussion of the obtained results and conclusions are reported in section 4.

## 2. Models and Dynamical Indicators

### 2.1. Network Model

We consider two sparsely coupled excitatory and inhibitory populations composed of *N*^(*e*)^ and *N*^(*i*)^ QIF neurons, respectively (Ermentrout and Kopell, 1986). The evolution equation for the membrane potentials $v_{j}^{\left(e\right)}$ and $v_{j}^{\left(i\right)}$ of the excitatory and inhibitory neurons can be written as:


(1a) $$\begin{array}{l} \tau_{m}{\dot{v}}_{j}^{\left(e\right)}={\left(v_{j}^{\left(e\right)}\right)}^{2}+I^{\left(e\right)}+2\tau_{m}[g^{\left(ee\right)}\underset{l|t_{l}^{\left(n\right)}<t}{\sum }\epsilon_{jl}^{\left(ee\right)}\delta \left(t-t_{l}^{\left(n\right)}\right)\\ \;-g^{\left(ei\right)}\underset{k|t_{k}^{\left(m\right)}<t}{\sum }\epsilon_{jk}^{\left(ei\right)}\delta \left(t-t_{k}^{\left(m\right)}\right)] \end{array}$$

(1b) $$\begin{array}{l} \tau_{m}{\dot{v}}_{j}^{\left(i\right)}={\left(v_{j}^{\left(i\right)}\right)}^{2}+I^{\left(i\right)}+2\tau_{m}[g^{\left(ie\right)}\underset{l|t_{l}^{\left(n\right)}<t}{\sum }\epsilon_{jl}^{\left(ie\right)}\delta \left(t-t_{l}^{\left(n\right)}\right)\\ \;-g^{\left(ii\right)}\underset{k|t_{k}^{\left(m\right)}<t}{\sum }\epsilon_{jk}^{\left(ii\right)}\delta \left(t-t_{k}^{\left(m\right)}\right)] \end{array}$$

where τ_*m*_ = 20 ms is the membrane time constant that we set identical for excitatory and inhibitory neurons, *I*^(*e*)^ (*I*^(*i*)^) is the external direct current (DC) acting on excitatory (inhibitory) population, *g*^(αβ)^ represents the synaptic coupling strengths between post synaptic neurons in the population α and pre synaptic ones in population β, with α, β ∈ {*e, i*}. The elements of the adjacency matrices $\epsilon_{jk}^{\left(\alpha \beta \right)}$ are equal to 1 (0) if a connection from a pre synaptic neuron *k* of population β toward a post synaptic neuron *j* of population α, exists (or not). Furthermore, $k_{j}^{\left(\alpha \beta \right)}=\underset{k}{\sum }\epsilon_{jk}^{\left(\alpha \beta \right)}$ is the number of pre synaptic neurons in the population β connected to neuron *j* in population α, or in other terms, its in-degree restricted to population β. The emission of the *n*-th spike emitted by neuron *l* of the population α occurs at time $t_{l}^{\left(n\right)}$ whenever the membrane potential $v_{l}^{\left(\alpha \right)}\left({t_{l}^{\left(n\right)}}^{-}\right)\to \infty$, while the reset mechanism is modeled by setting $v_{l}^{\left(\alpha \right)}\left({t_{l}^{\left(n\right)}}^{+}\right)\to -\infty$ immediately after the spike emission. The postsynaptic potentials are assumed to be δ-pulses and the synaptic transmissions to be instantaneous. The Equations (1) can be formally rewritten as


(2) $$\begin{array}{l} \tau_{m}{\dot{v}}_{j}^{\left(e\right)}={\left(v_{j}^{\left(e\right)}\right)}^{2}+i_{eff,j}^{\left(e\right)}\;,\;\tau_{m}{\dot{v}}_{j}^{\left(i\right)}={\left(v_{j}^{\left(i\right)}\right)}^{2}+i_{eff,j}^{\left(i\right)}\;; \end{array}$$

where $i_{eff,j}^{\left(e\right)}$ ($i_{eff,j}^{\left(i\right)}$) represents the instantaneous excitatory (inhibitory) effective currents, which include the external DC current and the synaptic currents due to the recurrent connections.

We consider the neurons within the excitatory and inhibitory populations as randomly connected, with in-degrees *k*^(αα)^ distributed according to a Lorentzian distribution


(3) $$\begin{array}{l} P\left(k^{\left(\alpha \alpha \right)}\right)=\frac{\Delta_{k}^{\left(\alpha \alpha \right)}}{{\left(k^{\left(\alpha \alpha \right)}-K^{\left(\alpha \alpha \right)}\right)}^{2}+{\Delta_{k}^{\left(\alpha \alpha \right)}}^{2}}\cdot \frac{1}{\pi } \end{array}$$

peaked at *K*^(αα)^ and with a half-width half-maximum (HWHM) $\Delta_{k}^{\left(\alpha \alpha \right)}$, this latter parameter measures the level of structural heterogeneity in each population. For simplicity, we set *K*^(*ee*)^ = *K*^(*ii*)^ ≡ *K*. Furthermore, we assume that also neurons from a population α are randomly connected to neurons of a different population β ≠ α. However, in this case, we consider no structural heterogeneity with in-degrees fixed to a constant value *K*^(*ei*)^ = *K*^(*ie*)^ = *K*. We have verified that by considering Erdös-Renyi distributed in-degrees *K*^(*ei*)^ and *K*^(*ie*)^ with average *K* does not modify the observed dynamical behavior.

The DC current and the synaptic coupling are rescaled with the median in degree as $I^{\left(\alpha \right)}=\sqrt{K}I_{0}^{\left(\alpha \right)}$ and $g^{\left(\alpha \beta \right)}=g_{0}^{\left(\alpha \beta \right)}/\sqrt{K}$, as done in previous studies to obtain a self-sustained balanced dynamics for *N* >> *K* >> 1 (van Vreeswijk and Sompolinsky, 1996; Renart et al., 2010; Litwin-Kumar and Doiron, 2012; Kadmon and Sompolinsky, 2015). The structural heterogeneity parameters are rescaled as $\Delta_{k}^{\left(\alpha \alpha \right)}=\Delta_{0}^{\left(\alpha \alpha \right)}\sqrt{K}$ in analogy to Erdös-Renyi networks. The choice of the Lorentzian distribution for the *k*^(αα)^ is needed in order to obtain an effective MF description for the microscopic dynamics (di Volo and Torcini, 2018; Bi et al., 2020) as detailed in the next section.

The microscopic activity can be analyzed by considering the inter-spike interval (ISI) distribution as characterized by the coefficient of variation *cv*_*i*_ for each neuron *i*, which is the ratio between the SD and the mean of the ISIs associated with the train of spikes emitted by the considered neuron. To characterize the macroscopic dynamics of each population α, we measure the average coefficient of variation $CV^{\left(\alpha \right)}=\overset{N^{\left(\alpha \right)}}{\underset{i=1}{\sum }}cv_{i}/N^{\left(\alpha \right)}$, the mean membrane potential $V^{\left(\alpha \right)}\left(t\right)=\overset{N^{\left(\alpha \right)}}{\underset{i=1}{\sum }}v_{i}^{\left(\alpha \right)}\left(t\right)/N^{\left(\alpha \right)}$, and the population firing rate *R*^(α)^(*t*), corresponding to the number of spikes emitted within the population α per unit of time and per neuron.

Furthermore, the level of coherence in the neural activity of the population α can be quantified in terms of the following indicator (Golomb, 2007),


(4) $$\begin{array}{l} \rho^{\left(\alpha \right)}={\left(\frac{\sigma_{V^{\left(\alpha \right)}}^{2}}{\sum_{i=1}^{N^{\left(\alpha \right)}}\sigma_{i}^{2}/N^{\left(\alpha \right)}}\right)}^{1/2} \end{array}$$

where $\sigma_{V^{\left(\alpha \right)}}$ is the SD of the mean membrane potential, $\sigma_{i}^{2}=\langle {\left(v_{i}^{\left(\alpha \right)}\right)}^{2}\rangle -\langle v_{i}^{\left(\alpha \right)}\rangle^{2}$ and 〈·〉 denotes a time average. A perfect synchrony corresponds to ρ^(α)^ = 1, while an asynchronous dynamics to a vanishing small $\rho^{\left(\alpha \right)}\approx O\left(1/\sqrt{N^{\left(\alpha \right)}}\right)$.

The frequencies associated with collective motions can be identified by measuring the power spectra *S*(ν) of the mean membrane potentials *V*(*t*) of the whole network. In the case of a periodic motion, the position of the main peak ν_*CO*_ represents the frequency of the COs, while for quasi-periodic motions, the spectrum is characterized by many peaks that can be obtained as a linear combination of two fundamental frequencies (ν_1_, ν_2_). The spectra obtained in the present case, always exhibit a continuous background due to the intrinsic fluctuations present in the balanced network. The power spectra have been obtained by calculating the temporal Fourier transform of *V*(*t*) sampled at time intervals of 10 ms. Time traces composed of 10,000 consecutive intervals have been considered to estimate the spectra, which are obtained at a frequency resolution of Δν = 0.01 Hz. Finally, the power spectra have been averaged over five independent realizations of the random network.

The network dynamics are integrated by employing an Euler scheme with time step *dt* = 0.0001 ms, while time averages and fluctuations are usually estimated on time intervals *T*_*s*_ ≃ 100 s, after discarding transients *T*_*t*_ ≃ 10 s. Usually, we consider networks composed of *N*^(*e*)^ = 10, 000 excitatory and *N*^(*i*)^ = 2, 500 inhibitory neurons.

### 2.2. Effective Neural Mass Model

In this sub-section, we derive a low dimensional effective neural mass formulation for the spiking network (Equation 1) by following Montbrió et al. (2015). In such an article, the authors obtained an exact MF model for a globally coupled heterogeneous population of QIF neurons by generalizing to neural systems using a reduction methodology previously developed for phase-coupled oscillators by Ott and Antonsen (2008). In particular, the neural mass model can be obtained by performing a rigorous mathematical derivation from the original spiking network in the limit *N* → ∞ by assuming that the heterogeneity present in the network, which can be either neuronal excitabilities or synaptic couplings, are distributed as Lorentzians. This MF reduction methodology gives rise to a neural mass model written in terms of only two collective variables: the mean membrane potential *V* and the instantaneous population rate *R*. For sufficiently large network size, the agreement between the simulation results and the neural mass model is impressive as shown in Montbrió et al. (2015) and in several successive publications.

The detailed derivation of the neural mass models from the corresponding spiking networks can be found in Montbrió et al. (2015), in this study, we limit to report its expression for a fully coupled homogeneous network of QIF neurons with synaptic couplings randomly distributed according to a Lorentzian:


(5a) $$\begin{array}{l} \tau_{m}Ṙ=2RV+\frac{\Gamma }{\pi }R \end{array}$$

(5b) $$\begin{array}{l} \tau_{m}\dot{V}=V^{2}+I+ḡ\tau_{m}R-{\left(\pi \tau_{m}R\right)}^{2} \end{array}$$

where ḡ is the median and Γ the HWHM of the Lorentzian distribution of the synaptic couplings.

Such formulation can be applied to the random sparse network studied in this article, in this paper. Indeed, as shown for a single sparse inhibitory population (di Volo and Torcini, 2018; Bi et al., 2020), the quenched disorder associated to the in-degree distribution can be rephrased in terms of random synaptic couplings. Namely, each neuron *i* in population α is subject to currents of amplitude $g_{0}^{\left(\alpha \beta \right)}k_{i}^{\left(\alpha \beta \right)}R^{\left(\beta \right)}/\left(\sqrt{K}\right)$ proportional to their in-degrees $k_{i}^{\left(\alpha \beta \right)}$, with β ∈ {*e, i*}. Therefore, we can consider the neurons as fully coupled but with random values of the couplings distributed as Lorentzian of median $g_{0}^{\left(\alpha \beta \right)}\sqrt{K}$ and HWHM $g_{0}^{\left(\alpha \beta \right)}\Delta_{0}^{\left(\alpha \beta \right)}$.

The neural mass model corresponding to the spiking network (Equation 1) can be written as follows:


(6a) $$\begin{array}{l} \tau_{m}{Ṙ}^{\left(e\right)}=R^{\left(e\right)}\left[2V^{\left(e\right)}+g_{0}^{\left(ee\right)}\frac{\Delta_{0}^{\left(ee\right)}}{\pi }\right] \end{array}$$

(6b) $$\begin{array}{l} \tau_{m}{\dot{V}}^{\left(e\right)}={\left[V^{\left(e\right)}\right]}^{2}-{\left[\pi R^{\left(e\right)}\tau_{m}\right]}^{2}\\ \;+\sqrt{K}\left[I_{0}^{\left(e\right)}+\left(g_{0}^{\left(ee\right)}R^{\left(e\right)}-g_{0}^{\left(ei\right)}R^{\left(i\right)}\right)\tau_{m}\right] \end{array}$$

(6c) $$\begin{array}{l} \tau_{m}{Ṙ}^{\left(i\right)}=R^{\left(i\right)}\left[2V^{\left(i\right)}+g_{0}^{\left(ii\right)}\frac{\Delta_{0}^{\left(ii\right)}}{\pi }\right] \end{array}$$

(6d) $$\begin{array}{l} \tau_{m}{\dot{V}}^{\left(i\right)}={\left[V^{\left(i\right)}\right]}^{2}-{\left[\pi R^{\left(i\right)}\tau_{m}\right]}^{2}\\ \;+\sqrt{K}\left[I_{0}^{\left(i\right)}+\left(g_{0}^{\left(ie\right)}R^{\left(e\right)}-g_{0}^{\left(ii\right)}R^{\left(i\right)}\right)\tau_{m}\right]\;; \end{array}$$

where we have set $\Delta_{0}^{\left(ei\right)}=\Delta_{0}^{\left(ie\right)}=0$, since we have assumed that the connections among neurons of different populations are random but with a fixed in-degree *K*^(*ei*)^ = *K*^(*ie*)^ = *K*.

#### 2.2.1. Stationary Solutions

The stationary solutions $\left\{{\bar{V}}^{\left(e\right)},{\bar{V}}^{\left(i\right)},{\bar{R}}^{\left(e\right)},{\bar{R}}^{\left(i\right)}\right\}$ of Equation (6) can be explicitly obtained for the mean membrane potentials as


(7) $$\begin{array}{l} {\bar{V}}^{\left(e\right)}=-\frac{g_{0}^{\left(ee\right)}\Delta_{0}^{\left(ee\right)}}{2\pi }\;,\;{\bar{V}}^{\left(i\right)}=-\frac{g_{0}^{\left(ii\right)}\Delta_{0}^{\left(ii\right)}}{2\pi }\;; \end{array}$$

while the instantaneous population rates are the solutions of the following quadratic system


(8a) $$\begin{array}{l} g_{0}^{\left(ee\right)}{\bar{R}}^{\left(e\right)}\tau_{m}-g_{0}^{\left(ei\right)}{\bar{R}}^{\left(i\right)}\tau_{m}=-I_{0}^{\left(e\right)}+\varepsilon \left\{{\left[\pi {\bar{R}}^{\left(e\right)}\tau_{m}\right]}^{2}-{\left[{\bar{V}}^{\left(e\right)}\right]}^{2}\right\} \end{array}$$

(8b) $$\begin{array}{l} g_{0}^{\left(ie\right)}{\bar{R}}^{\left(e\right)}\tau_{m}-g_{0}^{\left(ii\right)}{\bar{R}}^{\left(i\right)}\tau_{m}=-I_{0}^{\left(i\right)}+\varepsilon \left\{{\left[\pi {\bar{R}}^{\left(i\right)}\tau_{m}\right]}^{2}-{\left[{\bar{V}}^{\left(i\right)}\right]}^{2}\right\} \end{array}$$

where $\varepsilon =1/\sqrt{K}$ is a smallness parameter taking in to account finite in-degree corrections. It is interesting to notice that the parameters controlling the structural heterogeneity $\Delta_{0}^{\left(ii\right)}$ and $\Delta_{0}^{\left(ee\right)}$ fix the stationary values of the mean membrane potentials reported in Equation (7). The solutions of Equation (8) can be exactly obtained and the associated bifurcations analyzed by employing the software XPP AUTO developed for orbit continuation (Ermentrout, 2007).

For sufficiently large *K*, one can obtain analytic approximations of the solution of Equation (8) by expanding the population rates as follows:


(9) $$\begin{array}{l} {\bar{R}}^{\left(\alpha \right)}={\bar{R}}_{0}^{\left(\alpha \right)}+\varepsilon {\bar{R}}_{1}^{\left(\alpha \right)}+\varepsilon^{2}{\bar{R}}_{2}^{\left(\alpha \right)}+\varepsilon^{3}{\bar{R}}_{3}^{\left(\alpha \right)}+\ldots \;\alpha \in \left\{e,i\right\}, \end{array}$$

by inserting these expressions in Equation (8), and finally by solving order by order in ε.

The solutions at any order can be written as follows:


(10) $$\begin{array}{l} {\bar{R}}_{k}^{\left(e\right)}\tau_{m}=\frac{N_{k}^{\left(e\right)}g_{0}^{\left(ii\right)}-N_{k}^{\left(i\right)}g_{0}^{\left(ei\right)}}{g_{0}^{\left(ei\right)}g_{0}^{\left(ie\right)}-g_{0}^{\left(ee\right)}g_{0}^{\left(ii\right)}},\\ {\bar{R}}_{k}^{\left(i\right)}\tau_{m}=\frac{N_{k}^{\left(e\right)}g_{0}^{\left(ie\right)}-N_{k}^{\left(i\right)}g_{0}^{\left(ee\right)}}{g_{0}^{\left(ei\right)}g_{0}^{\left(ie\right)}-g_{0}^{\left(ee\right)}g_{0}^{\left(ii\right)}}; \end{array}$$

where,


(11a) $$\begin{array}{l} N_{0}^{\left(\alpha \right)}=I_{0}^{\left(\alpha \right)},N_{1}^{\left(\alpha \right)}={\left[{\bar{V}}^{\left(\alpha \right)}\right]}^{2}-{\left[\pi {\bar{R}}_{0}^{\left(\alpha \right)}\tau_{m}\right]}^{2} \end{array}$$

(11b) $$\begin{array}{l} N_{2j}^{\left(\alpha \right)}=-2{\left[\pi \tau_{m}\right]}^{2}\overset{j}{\underset{k=1}{\sum }}\left[{\bar{R}}_{k-1}^{\left(\alpha \right)}{\bar{R}}_{2j-k}^{\left(\alpha \right)}\right] \end{array}$$

(11c) $$\begin{array}{l} \;N_{2j+1}^{\left(\alpha \right)}=-2{\left[\pi \tau_{m}\right]}^{2}\left\{\left[\overset{j}{\underset{k=1}{\sum }}{\bar{R}}_{k-1}^{\left(\alpha \right)}{\bar{R}}_{2j+1-k}^{\left(\alpha \right)}\right]+\frac{1}{2}{\left[{\bar{R}}_{j}^{\left(\alpha \right)}\right]}^{2}\right\}\\ \text{for }j\geq 1 \end{array}$$

The systems (Equation 10) with parameters given by Equation (11) can be resolved recursively for any order and the final solution obtained from the expression (Equation 9). The zeroth-order approximation, valid in the limit *K* → ∞, corresponds to the usual solution found for rate models in the balanced state (van Vreeswijk and Sompolinsky, 1996; Rosenbaum and Doiron, 2014), such solution is physical whenever one of the following inequalities is satisfied


(12) $$\begin{array}{l} \frac{I_{0}^{\left(e\right)}}{I_{0}^{\left(i\right)}}>\frac{g_{0}^{\left(ei\right)}}{g_{0}^{\left(ii\right)}}>\frac{g_{0}^{\left(ee\right)}}{g_{0}^{\left(ie\right)}}\;,\;\frac{I_{0}^{\left(e\right)}}{I_{0}^{\left(i\right)}}<\frac{g_{0}^{\left(ei\right)}}{g_{0}^{\left(ii\right)}}<\frac{g_{0}^{\left(ee\right)}}{g_{0}^{\left(ie\right)}}\;; \end{array}$$

which ensure the positive sign of ${\bar{R}}_{0}^{\left(e\right)}$ and ${\bar{R}}_{0}^{\left(i\right)}$. The zeroth-order solution does not depend on the structural heterogeneity, since the ratio Δ^(αα)^/*K* vanishes in the limit *K* → ∞. It should be stressed that this ratio does not correspond to the coefficient of variation introduced in Landau et al. (2016) to characterize the in-degree distribution. This is because we are considering a Lorentzian distribution, where the average and the SD are not even defined. Moreover, already the first-order corrections depend on $\Delta_{0}^{\left(\alpha \alpha \right)}$.

To characterize the level of balance in the system, one usually estimates the values of the effective input currents $i_{eff,j}^{\left(e\right)}$ and $i_{eff,j}^{\left(i\right)}$ driving the neuron dynamics. These at a population level can be rewritten as


(13) $$\begin{array}{l} I_{eff}^{\left(e\right)}=\sqrt{K}\left[I_{0}^{\left(e\right)}+\tau_{m}\left(g_{0}^{\left(ee\right)}R^{\left(e\right)}-g_{0}^{\left(ei\right)}R^{\left(i\right)}\right)\right],\\ I_{eff}^{\left(i\right)}=\sqrt{K}\left[I_{0}^{\left(i\right)}+\tau_{m}\left(g_{0}^{\left(ie\right)}R^{\left(e\right)}-g_{0}^{\left(ii\right)}R^{\left(i\right)}\right)\right]. \end{array}$$

In a balanced state, these quantities should not diverge with *K*, instead, they should approach some constant value. For an asynchronous state we can estimate analytically, within our MF formulation, the values of the effective currents in the limit *K* → ∞. These read as


(14) $$\begin{array}{l} I_{a}^{\left(e\right)}=\tau_{m}\left[g_{0}^{\left(ee\right)}{\bar{R}}_{1}^{\left(e\right)}-g_{0}^{\left(ei\right)}{\bar{R}}_{1}^{\left(i\right)}\right],\\ I_{a}^{\left(i\right)}=\tau_{m}\left[g_{0}^{\left(ie\right)}{\bar{R}}_{1}^{\left(e\right)}-g_{0}^{\left(ii\right)}{\bar{R}}_{1}^{\left(i\right)}\right]\;. \end{array}$$

It should be noticed that these asymptotic values depend on the first-order corrections to the balanced solution (Equation 10). Therefore, they depend not only on the synaptic couplings $g_{0}^{\left(\alpha \beta \right)}$ and on the external DC currents but also on the parameters $\Delta_{0}^{\left(\alpha \alpha \right)}$ controlling the structural heterogeneities.

Depending on the parameter values, the currents $I_{a}^{\left(\alpha \right)}$ can be positive or negative, thus, indicating a balanced dynamics where most parts of the neurons are supra or below the threshold, respectively. Usually, in order to obtain a stationary state characterized by a low rate and a Poissonian statistic, as observed in the cortex, one assumes that the excitation and inhibition nearly cancel. So that the mean membrane potential remains slightly below the threshold, and the neurons can fire occasionally due to the input current fluctuations (van Vreeswijk and Sompolinsky, 1996; Brunel, 2000). However, as pointed out in Lerchner et al. (2006), this is not the only possible scenario for a balanced state. In particular, the authors have developed a self-consistent MF theory for balanced Erdös-Renyi networks made of heterogeneous Leaky Integrate-and-Fire (LIF) neurons. In this context, they have shown that Poisson-like dynamics are visible only at intermediate synaptic couplings. While mean driven dynamics are expected for low couplings, and at large couplings bursting behaviors appear in the balanced network. Recently, analogous dynamical behaviors have been reported also for a purely inhibitory heterogeneous LIF network (Angulo-Garcia et al., 2017). These findings are consistent with the results in Lerchner et al. (2006), where the inhibition is indeed predominant in the balanced regime.

#### 2.2.2. Lyapunov Analysis

To analyze the linear stability of generic solutions of Equation (6), we have estimated the corresponding Lyapunov spectrum (LS) {λ_*k*_} (Pikovsky and Politi, 2016). This can be done by considering the time evolution of the tangent vector δ = {δ*R*^(*e*)^, δ*V*^(*e*)^, δ*R*^(*i*)^, δ*V*^(*i*)^}, that is ruled by the linearization of the Equation (6), namely


(15a) $$\begin{array}{l} \tau_{m}\delta {Ṙ}^{\left(e\right)}=\left[2V^{\left(e\right)}+g_{0}^{\left(ee\right)}\frac{\Delta_{0}^{\left(ee\right)}}{\pi }\right]\delta R^{\left(e\right)}+2R^{\left(e\right)}\delta V^{\left(e\right)} \end{array}$$

(15b) $$\begin{array}{l} \tau_{m}\delta {\dot{V}}^{\left(e\right)}=2V^{\left(e\right)}\delta V^{\left(e\right)}-2{\left(\pi \tau_{m}\right)}^{2}R^{\left(e\right)}\delta R^{\left(e\right)}\\ \;+\sqrt{K}\tau_{m}\left[g_{0}^{\left(ee\right)}\delta R^{\left(e\right)}-g_{0}^{\left(ei\right)}\delta R^{\left(i\right)}\right] \end{array}$$

(15c) $$\begin{array}{l} \tau_{m}\delta {Ṙ}^{\left(i\right)}=\left[2V^{\left(i\right)}+g_{0}^{\left(ii\right)}\frac{\Delta_{0}^{\left(ii\right)}}{\pi }\right]\delta R^{\left(i\right)}+2R^{\left(i\right)}\delta V^{\left(i\right)} \end{array}$$

(15d) $$\begin{array}{l} \tau_{m}\delta {\dot{V}}^{\left(i\right)}=2V^{\left(i\right)}\delta V^{\left(i\right)}-2{\left(\pi \tau_{m}\right)}^{2}R^{\left(i\right)}\delta R^{\left(i\right)}\\ \;+\sqrt{K}\tau_{m}\left[g_{0}^{\left(ie\right)}\delta R^{\left(e\right)}-g_{0}^{\left(ii\right)}\delta R^{\left(i\right)}\right]\;. \end{array}$$

In this case, the LS is composed of four Lyapunov exponents (LEs) {λ_*k*_} with *k* = 1, …, 4, which quantify the average growth rates of infinitesimal perturbations along the orthogonal manifolds. The LEs can be estimated as follows:


(16) $$\begin{array}{l} \lambda_{k}=\underset{t\to \infty }{\lim}\frac{1}{t}\log\frac{\left|\delta_{k}\left(t\right)\right|}{\left|\delta_{k}\left(0\right)\right|}\;, \end{array}$$

where the tangent vectors **δ**_*k*_ are maintained ortho-normal during the time evolution by employing a standard technique introduced in Benettin et al. (1980). The autonomous system will be chaotic for λ_1_ > 0, while a periodic (two frequency quasi-periodic) dynamics will be characterized by λ_1_ = 0 (λ_1_ = λ_2_ = 0) and a fixed point by λ_1_ < 0.

In order to estimate the LS for the neural mass model, we have integrated the direct and tangent space evolution with a Runge-Kutta 4th order integration scheme with *dt* = 0.01 ms, for a duration of 200 s, after discarding a transient of 10 s.

#### 2.2.3. Linear Stability of Stationary Solutions

The linear stability of the stationary solutions $\left\{{\bar{V}}^{\left(e\right)},{\bar{V}}^{\left(i\right)},{\bar{R}}^{\left(e\right)},{\bar{R}}^{\left(i\right)}\right\}$ can be analyzed by solving the eigenvalue problem for the linear Equations (15) estimated for stationary values of the mean membrane potentials and of the population firing rates. This approach gives rise to a fourth-order characteristic polynomial of the complex eigenvalues $\Lambda^{\left(k\right)}=\Lambda_{R}^{\left(k\right)}+i\Lambda_{I}^{\left(k\right)}$ with *k* = 1, …, 4. The stability of the fixed point is controlled by the maximal $\Lambda_{R}^{\left(k\right)}$, whenever it is positive (negative), the stationary solution is unstable (stable). The nature of the fixed point is determined by $\Lambda_{I}^{\left(k\right)}$, if the imaginary parts of the eigenvalues are all zero, we have a node, otherwise a focus. Due to the fact that the coefficients of the characteristic polynomial are real, the eigenvalues are real or if complex they appear in complex conjugates couples $\Lambda_{R}^{\left(j\right)}\pm i\Lambda_{I}^{\left(k\right)}$. Therefore, the relaxation toward the fixed point is characterized by one or two frequencies $\nu_{k}=\Lambda_{I}^{\left(k\right)}/\left(2\pi \right)$. These latter quantities, as discussed in detail in the following, can give good predictions for the frequencies ν_*CO*_ of fluctuation driven COs observable for the same parameters in the network dynamics.

In the limit *K* >> 1, we can approximate the linear stability (Equations 15) as follows:


(17a) $$\begin{array}{l} \tau_{m}\delta {Ṙ}^{\left(e\right)}=2{\bar{R}}_{0}^{\left(e\right)}\delta V^{\left(e\right)} \end{array}$$

(17b) $$\begin{array}{l} \tau_{m}\delta {\dot{V}}^{\left(e\right)}=\sqrt{K}\tau_{m}\left[g_{0}^{\left(ee\right)}\delta R^{\left(e\right)}-g_{0}^{\left(ei\right)}\delta R^{\left(i\right)}\right] \end{array}$$

(17c) $$\begin{array}{l} \tau_{m}\delta {Ṙ}^{\left(i\right)}=2{\bar{R}}_{0}^{\left(i\right)}\delta V^{\left(i\right)} \end{array}$$

(17d) $$\begin{array}{l} \tau_{m}\delta {\dot{V}}^{\left(i\right)}=\sqrt{K}\tau_{m}\left[g_{0}^{\left(ie\right)}\delta R^{\left(e\right)}-g_{0}^{\left(ii\right)}\delta R^{\left(i\right)}\right]\;; \end{array}$$

where we have considered the zeroth-order approximation for the population rates ${\bar{R}}_{0}^{\left(e\right)}$ and ${\bar{R}}_{0}^{\left(i\right)}$.

In this case, the complex eigenvalues Λ^(*k*)^ are given by the following expression:


(18) $$\begin{array}{l} \;{\left[\Lambda^{\left(k\right)}\right]}^{2}=\frac{\sqrt{K}}{\tau_{m}}[\left(g_{0}^{\left(ee\right)}{\bar{R}}_{0}^{\left(e\right)}-g_{0}^{\left(ii\right)}{\bar{R}}_{0}^{\left(i\right)}\right)\\ \pm \sqrt{{\left(g_{0}^{\left(ee\right)}{\bar{R}}_{0}^{\left(e\right)}+g_{0}^{\left(ii\right)}{\bar{R}}_{0}^{\left(i\right)}\right)}^{2}-4g_{0}^{\left(ei\right)}g_{0}^{\left(ie\right)}{\bar{R}}_{0}^{\left(e\right)}{\bar{R}}_{0}^{\left(i\right)}}]\;. \end{array}$$

From Equation (18), it is evident that Λ^(*k*)^ ∝ (*K*)^1/4^, and by assuming $I_{0}^{\left(i\right)}\propto I_{0}^{\left(e\right)}$, as we will do in this study, we also have that $\Lambda^{\left(k\right)}\propto {\left(I_{0}^{\left(e\right)}\right)}^{1/2}$. Therefore, for a focus solution, we will have the following scaling relation for the relaxation frequencies for sufficiently large *K*:


(19) $$\begin{array}{l} \nu_{k}^{R}=\frac{\Lambda_{I}^{\left(k\right)}}{2\pi }\propto \sqrt{I_{0}^{\left(e\right)}K^{1/2}}\;; \end{array}$$

This scaling is analogous to that found for purely inhibitory QIF networks in di Volo and Torcini (2018). In van Vreeswijk and Sompolinsky (1996), it has been found that the eigenvalues, characterizing the stability of the asynchronous state, scale proportionally to $\sqrt{K}$, therefore, the convergence (divergence) from the stationary stable (unstable) solution is somehow slower with *K* in our model. This is due to the presence in our MF of an extra macroscopic variable, the mean membrane potential, with respect to the usual rate models.

## 3. Results

### 3.1. Phase Diagrams

In this sub-section, we will investigate the possible dynamical regimes emerging in our model by employing its neural mass formulation. In particular, the dynamics of the neural mass model (Equation 6) take place in a four-dimensional space {*R*^(*e*)^, *V*^(*e*)^, *R*^(*i*)^, *V*^(*i*)^} and it depends on nine parameters, namely on the four synaptic coupling strengths $\left\{g_{0}^{\left(ee\right)},g_{0}^{\left(ei\right)},g_{0}^{\left(ii\right)},g_{0}^{\left(ie\right)}\right\}$, the two external stimulation currents $\left\{I_{0}^{\left(e\right)},I_{0}^{\left(i\right)}\right\}$, the median in-degree *K*, and the HWHM of the two distributions of the in-degrees $\left\{\Delta_{0}^{\left(ee\right)},\Delta_{0}^{\left(ii\right)}\right\}$.

However, in order to reduce the space of parameters to investigate and at the same time to satisfy the inequalities (Equation 12) required for the existence of a balanced state in the large *K* limit, we fix the inhibitory DC current as $I_{0}^{\left(i\right)}=I_{0}^{\left(e\right)}/1.02$ and the synaptic couplings as $g_{0}^{\left(ee\right)}=0.27$, $g_{0}^{\left(ii\right)}=0.953939$, $g_{0}^{\left(ie\right)}=0.3$, and $g_{0}^{\left(ei\right)}=0.96286$ analogously to what was done in Monteforte and Wolf (2010). Therefore, we are left with four control parameters, namely $\Delta_{0}^{\left(ee\right)}$, $\Delta_{0}^{\left(ii\right)}$, $I_{0}^{\left(e\right)}$, and *K*, that we will vary to investigate the possible dynamical states.

**Figure 1:**
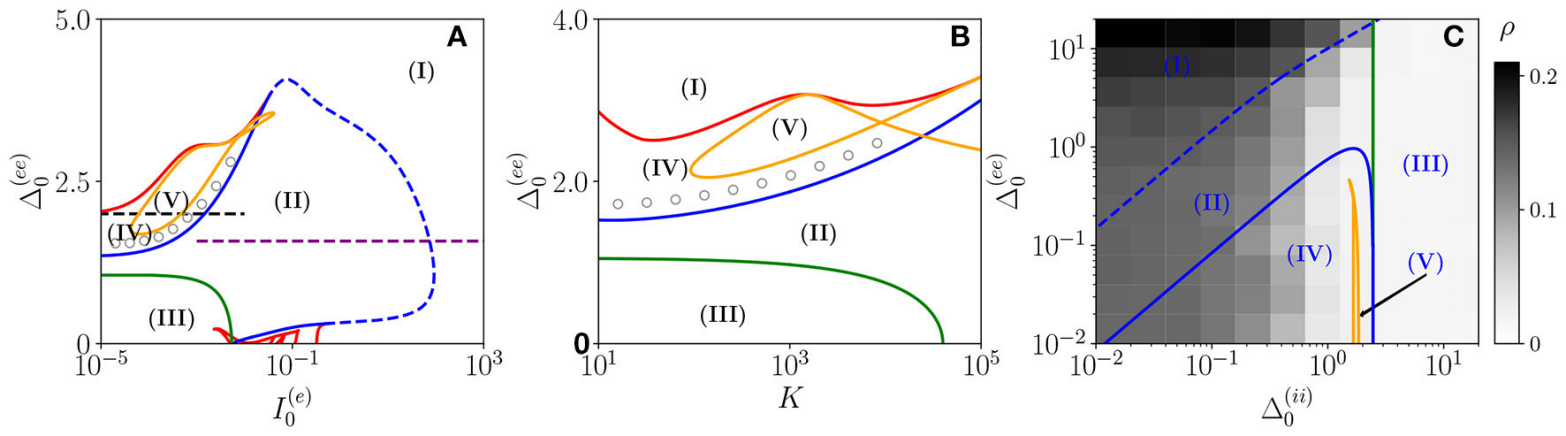
Bifurcation diagrams of the neural mass model. The bifurcation diagrams concern the dynamical state exhibited by the excitatory population in the bidimensional parameter spaces $\left(I_{0}^{\left(e\right)},\Delta_{0}^{\left(ee\right)}\right)$ **(A)**, $\left(K,\Delta_{0}^{\left(ee\right)}\right)$ **(B)**, and $\left(\Delta_{0}^{\left(ii\right)},\Delta_{0}^{\left(ee\right)}\right)$ **(C)**. The regions marked by Roman numbers correspond to the following collective solutions: (I) an unstable focus; (II) a stable focus coexisting with an unstable limit cycle (LC); (III) a stable node; (IV) an unstable focus coexisting with a stable LC; (V) a chaotic dynamics. The green solid line separates the regions with a stable node (III) and a stable focus (II). The blue solid (dashed) curve is a line of super-critical (sub-critical) Hopf bifurcations (HBs), and the red one of saddle-node (SN) bifurcations of LCs. The yellow curve denotes the period doubling (PD) bifurcation lines. In **(C)**, we also report the coherence indicator ρ^(*e*)^ (Equation 4) estimated from the network dynamics with *N*^(*e*)^ = 10,000 and *N*^(*i*)^ = 2,500. The dashed lines in **(A)** indicate the parameter cuts we will consider in Figures 4, 5 (black) and Figure 7 (purple), while the open circles in **(A,B)** denote the set of parameters employed in Figure 11. In the three panels, the inhibitory DC current and the synaptic couplings are fixed to $I_{0}^{\left(i\right)}=I_{0}^{\left(e\right)}/1.02$, $g_{0}^{\left(ee\right)}=0.27$, $g_{0}^{\left(ii\right)}=$0.953939, $g_{0}^{\left(ie\right)}=0.3$, $g_{0}^{\left(ei\right)}=$0.96286; other parameters: **(A)** *K*=1,000, $\Delta_{0}^{\left(ii\right)}=0.3$, **(B)** $I_{0}^{\left(e\right)}=$0.001, $\Delta_{0}^{\left(ii\right)}=0.3$, **(C)** *K*= 1,000, and $I_{0}^{\left(e\right)}=0.1$.

Three bidimensional bifurcation diagrams for the neural mass model (Equation 6) are reported in Figure 1 for the couple of parameters $\left(I_{0}^{\left(e\right)},\Delta_{0}^{\left(ee\right)}\right)$, $\left(K,\Delta_{0}^{\left(ee\right)}\right)$, and $\left(\Delta_{0}^{\left(ii\right)},\Delta_{0}^{\left(ee\right)}\right)$. From the bifurcation analysis, we have identified five different dynamical states for the excitatory population: namely, (I) an unstable focus; (II) a stable focus coexisting with an unstable limit cycle (LC); (III) a stable node; (IV) a stable limit cycle coexisting with an unstable focus; and (V) a chaotic regime. For the analysis reported in the following, it is important to remark that the stable foci are usually associated with four complex eigenvalues arranged in complex conjugate couples, therefore, the relaxation toward a stable focus is characterized by two frequencies (ν_1_, ν_2_) corresponding to the complex parts of the eigenvalues. In region (III), the macroscopic fixed point is characterized by two real eigenvalues and a couple of complex conjugated ones. Thus, the relaxation toward the macroscopic node is, in this case, guided by a single relaxation frequency. The inhibitory population, reveals the same bifurcation structure as the excitatory one, apart from an important difference: the inhibitory population never displays stable nodes. Therefore, the region (III) for the inhibitory population is also a region of type (II).

As shown in Figures 1A,B, for fixed $\Delta_{0}^{\left(ii\right)}$ and for low values of the structural heterogeneity $\Delta_{0}^{\left(ee\right)}$ and of the excitatory DC current $I_{0}^{\left(e\right)}$, one observes a stable node (III) that becomes a stable focus (II) by increasing $\Delta_{0}^{\left(ee\right)}$, these transitions are signaled as green solid lines in Figure 1. By further increasing the degree of heterogeneity $\Delta_{0}^{\left(ee\right)}$, the stable focus gives rise to COs (IV) *via* a super-critical HB (blue solid lines). Depending on the values of *K* and $I_{0}^{\left(e\right)}$, one can have the emergence of chaotic behaviors (V) *via* a period doubling (PD) cascade (yellow solid lines). For sufficiently large $\Delta_{0}^{\left(ee\right)}$, the COs disappear *via* a saddle-node (SN) bifurcation of limit cycles (LC) (red solid lines) and above the SN line, the only remaining solution is an unstable focus (I).

As shown in Figure 1A, for fixed structural heterogeneities, the increase of $I_{0}^{\left(e\right)}$ leads to the disappearance of the stable focus (II) *via* a sub-critical HB (dashed blue line). The dependence of the observed MF solutions on the in-degree *K* is reported in Figure 1B for a current $I_{0}^{\left(e\right)}=0.001$, and it is not particularly dramatic apart from for the emergence of a chaotic region (V) from a CO regime (IV).

In order to observe the emergence of COs (IV) from the destabilization of a node solution (III), we should vary the structural inhibitory heterogeneity $\Delta_{0}^{\left(ii\right)}$, as shown in Figure 1C. Indeed, for sufficiently low $\Delta_{0}^{\left(ii\right)}$ and $\Delta_{0}^{\left(ee\right)}$, we can observe a super-critical bifurcation line from a node to a stable LC. From this analysis, it emerges that the excitatory heterogeneity has an opposite effect with respect to the inhibitory one, indeed by increasing $\Delta_{0}^{\left(ee\right)}$, the value of ρ^(*e*)^ increases indicating the presence of more synchronized COs. This effect is due to the fact that the increase of $\Delta_{0}^{\left(ee\right)}$ leads to more and more neurons with large $k_{j}^{\left(ee\right)}>>K$, therefore, receiving higher and higher levels of recurrent excitation. These neurons are definitely supra-threshold and drive the activity of the network toward coherent behaviors.

In order to understand the limits of our MF formulation, it is of particular interest to compare the network simulations with the MF phase diagram. To this aim, we report in Figure 1C, the coherence indicator ρ^(*e*)^ (Equation 4) estimated from the network dynamics. The indicator ρ^(*e*)^ reveals that no COs are present in the region (III), where the MF displays a stable node, however, COs emerge in all the other MF regimes for sufficiently low $\Delta_{0}^{\left(ii\right)}<1$. The presence of COs is expected from the MF analysis only in the regions (IV) and (V), but neither in (II) where the MF forecasts the existence of a stable focus nor in (I) where no stable solutions are envisaged. The origin of the discrepancies among the MF and the network simulations in the region (II) is due to the fact that the considered neural mass neglects the dynamical fluctuations in the input currents present in the original networks, which can give rise to noise induced COs (Goldobin et al., 2021). However, as shown in di Volo and Torcini (2018) and Bi et al. (2020) for purely inhibitory populations, the analysis of the neural mass model can still give relevant information on the network dynamics. In particular, the frequencies of the fluctuation induced COs observable in the network simulations can be well estimated from the frequencies (ν_1_, ν_2_) of the relaxation oscillations toward the stable MF focus. The lack of agreement between MF and network simulations in the region (I) is due to finite size effects, indeed in this case, the system tends to fully synchronize. Therefore, in the network, one observes highly synchronized COs characterized by population firing rates that diverge for increasing *K* and *N* and the MF is unable to reproduce these unrealistic solutions (Montbrió et al., 2015).

On the basis of these observations, we can classify the COs observable in the network in three different types accordingly to the corresponding MF solutions: O_P_, when in the MF we observe periodic, quasi-periodic, or chaotic collective solutions in regions (IV) and (V); O_F_, when the MF displays relaxation oscillations toward the stable focus in regions (II) and (III), that in the sparse network become noise sustained oscillations due to fluctuations in the input currents; O_S_, when the MF fully synchronizes as in region (I).

In the following sub-sections, we will analyze the macroscopic dynamics of the E-I network of QIF neurons in order to test the predictions of the effective neural mass mode for asynchronous and coherent dynamics. In this latter case, we will focus on the three types of identified COs: namely, O_P_, O_F_, and O_S_. These can manifest as periodic, quasi-periodic, and chaotic solutions as we will see by examining two main scenarios indicated as dashed horizontal lines in Figure 1A corresponding to the transition to chaos (black dashed line) and the emergence of abnormal synchronization from a stable focus (purple dashed line).

### 3.2. Asynchronous Regimes

**Figure 2:**
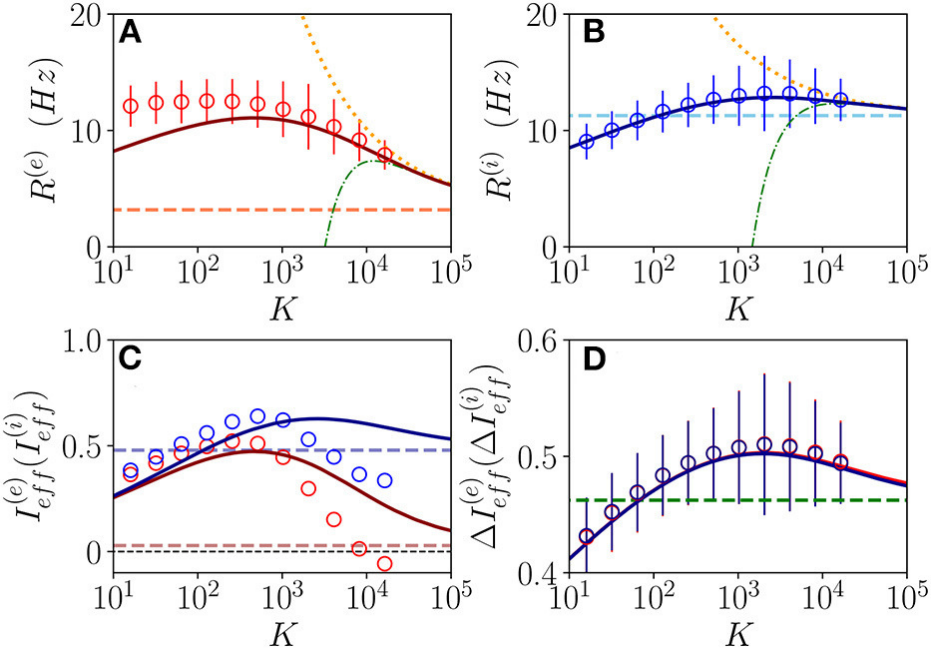
Asynchronous dynamics: Instantaneous population rate *R*^(*e*)^ (*R*^(*i*)^) of excitatory (inhibitory) neurons in function of the median in-degree *K* are shown in **(A,B)**. The effective input currents $I_{eff}^{\left(e\right)}$ ($I_{eff}^{\left(i\right)}$) given by Equations (13) are reported in **(C)** and the fluctuations of the input currents $\Delta I_{eff}^{\left(e\right)}$ ($\Delta I_{eff}^{\left(i\right)}$), as obtained from Equations (20), in **(D)**. Red (blue) color refers to excitatory inhibitory population. The solid continuous lines represent the value obtained by employing the exact MF solutions ${\bar{R}}^{\left(x\right)}$ of Equation (8), the dotted (dash-dotted) lines correspond to the first (second) order approximation ${\bar{R}}_{0}^{\left(x\right)}+\varepsilon {\bar{R}}_{1}^{\left(x\right)}$ (${\bar{R}}_{0}^{\left(x\right)}+\varepsilon {\bar{R}}_{1}^{\left(x\right)}+\varepsilon^{2}{\bar{R}}_{2}^{\left(x\right)}$) and the dashed horizontal lines to the zeroth-order one ${\bar{R}}_{0}^{\left(x\right)}$ in **(A,B,D)**, and to $I_{a}^{\left(x\right)}$ in **(C)** with *x* = *e, i*. The circles correspond to data obtained from numerical simulations of *N*^(*e*)^ = *N*^(*i*)^ = 10,000 neurons for *K* < 4,096, *N*^(*e*)^ = *N*^(*i*)^ = 20,000 for *K* = 4,096,8,192 and *N*^(*e*)^ = *N*^(*i*)^ = 30,000 for *K* > 8, 192, averaging the population rates over a window of *T* = 40 *s*, after discarding a transient of *T* = 60 *s*. The error bars in **(A,B)** are obtained as the SD (over the time window *T*) of the population rates, while the average CV of neurons is around 0.15 for all the reported simulations. Synaptic couplings and the ratio between the currents are fixed as stated in sub-section 3.1, other parameters are $\Delta_{0}^{\left(ii\right)}=1$, $\Delta_{0}^{\left(ee\right)}=2.5$, and $I_{0}^{\left(e\right)}=0.2$. The values of the asymptotic solutions (dashed lines) are : in **(A,B)** ${\bar{R}}_{0}^{\left(e\right)}=3.18$ Hz and ${\bar{R}}_{0}^{\left(i\right)}=11.28$ Hz, respectively; in **(C)** $I_{a}^{\left(e\right)}=$ 0.0284 and $I_{a}^{\left(i\right)}≃0.4791$; in **(D)** $\Delta I_{eff}^{\left(e\right)}=$ 0.4623 and $\Delta I_{eff}^{\left(i\right)}=$ 0.4593.

We will first consider a situation where the network dynamics remains asynchronous for any value of the median in-degree *K*, this occurs for sufficiently high structural inhibitory heterogeneities $\Delta_{0}^{\left(ii\right)}$ and external DC currents as shown in Figures 1B,C for E-I networks and as reported in di Volo and Torcini (2018) for purely inhibitory populations. If the population dynamics are asynchronous, we expect that at an MF level, the system will converge toward a stationary state corresponding to a stable equilibrium. Therefore, we have compared the results of the network simulations with the stationary rates $\left({\bar{R}}^{\left(e\right)},{\bar{R}}^{\left(i\right)}\right)$ solutions of Equation (6). As shown in Figures 2A,B, the macroscopic activity of the excitatory and inhibitory populations is well reproduced by the fixed point solutions (Equation 8) in a wide range of values of the in-degrees 10 ≤ *K* ≤ 10^4^. This is particularly true for the inhibitory population, while at low *K* < 100, the excitatory firing rate is slightly underestimated by the macroscopic solution ${\bar{R}}^{\left(e\right)}$. Due to our choice of parameters, the average inhibitory firing rate is larger than the excitatory one for *K* > 100. This is consistent with experimental data reported for the barrel cortex of behaving mice (Gentet et al., 2010) and other cortical areas (Mongillo et al., 2018). Moreover, the rates have a non-monotonic behavior with *K* with a maximum at *K* ≃ 450 (*K* ≃ 2,500) for excitatory (inhibitory) neurons. As expected, the balanced state solutions ${\bar{R}}_{0}^{\left(e\right)}=3.18$ Hz and ${\bar{R}}_{0}^{\left(i\right)}≃11.28$ Hz (dashed horizontal lines) are approached only for sufficiently large *K* >> 1. In Figures 1A,B are reported also the first (second) order approximation ${\bar{R}}_{0}^{\left(e\right)}+\varepsilon {\bar{R}}_{1}^{\left(e\right)}$ (${\bar{R}}_{0}^{\left(e\right)}+\varepsilon {\bar{R}}_{1}^{\left(e\right)}+\varepsilon^{2}{\bar{R}}_{2}^{\left(e\right)}$) given by Equation (10). These approximations reproduce quite well the complete solutions already at *K* ≥ 10^4^.

Let us now consider the effective input currents (Equation 13), these are reported in Figure 2C vs. the median in-degree. As expected, for increasing *K*, the MF estimations of the effective currents (solid lines) converge to the asymptotic values $I_{a}^{\left(e\right)}≃$ 0.0284 and $I_{a}^{\left(i\right)}≃$ 0.4791 (dashed lines) for our choice of parameters. For the excitatory population, the asymptotic value of the effective input current is essentially zero, while for the inhibitory population it is positive. These results suggest that for the considered choice of parameters the dynamics of both populations will be balanced, since the quantities $I_{a}^{\left(e\right)}$ and $I_{a}^{\left(i\right)}$ do not diverge with *K*, however, at a macroscopic level, the excitatory population will be at the threshold, while the inhibitory one will be supra-threshold. For comparison, we have estimated $I_{eff}^{\left(\alpha \right)}$ also from the direct the network simulations (circles) for 16 ≤ *K* ≤ 16,384. These estimations disagree with the MF results already for *K* > 1,000. This is despite the fact that the population firing rates in the network are very well captured by the MF estimations at large *K*, as shown in Figures 2A,B. These large differences in the effective input currents are the effect of small discrepancies at the level of firing rates enhanced by the multiplicative factor $\sqrt{K}$ appearing in Equations (13). However, from the network simulations, we observe that the effective currents approach values smaller than the asymptotic ones $I_{a}^{\left(e\right)}$ and $I_{a}^{\left(i\right)}$ obtained from the neural mass model. In particular, despite the fact that from finite *K* simulations, it is difficult to extrapolate the asymptotic behaviors, it appears that $I_{eff}^{\left(e\right)}$ approaches a small negative value for *K* >> 1, while $I_{eff}^{\left(i\right)}$ converges to some finite positive value. In the following, we will see the effect of these different behaviors on microscopic dynamics. The origin of the reported discrepancies should be related to the presence of current fluctuations in the network that are neglected in the MF formulation.

The relevance of the current fluctuations for the network dynamics can be appreciated by estimating their amplitudes within a Poissonian approximation, as follows


(20) $$\begin{array}{l} \Delta I_{eff}^{\left(e\right)}=\sqrt{\tau_{m}\left[{\left(g_{0}^{\left(ee\right)}\right)}^{2}R^{\left(e\right)}+{\left(g_{0}^{\left(ei\right)}\right)}^{2}R^{\left(i\right)}\right]}\;\\ \Delta I_{eff}^{\left(i\right)}=\sqrt{\tau_{m}\left[{\left(g_{0}^{\left(ie\right)}\right)}^{2}R^{\left(e\right)}+{\left(g_{0}^{\left(ii\right)}\right)}^{2}R^{\left(i\right)}\right]} \end{array}$$

These have been evaluated by assuming that each neuron receives on average *K* excitatory and inhibitory spike trains characterized by Poissonian statistics with average rates *R*^(*e*)^ and *R*^(*i*)^. However, we have neglected in the above estimation the variability of the in-degrees of each neuron. As shown in Figure 2D, these fluctuations are essentially identical for excitatory and inhibitory neurons and coincide with the MF results. In the limit *K* >> 1, they converge to the asymptotic values $\Delta I_{eff}^{\left(e\right)}≃$ 0.4623 and $\Delta I_{eff}^{\left(i\right)}≃$ 0.4593 (green dashed lines). It is evident that already for *K* > 1,000, the amplitudes of the fluctuations are of the same order or larger than the effective input currents. Thus, suggesting that the fluctuations have indeed a relevant role in determining the network dynamics and that one would observe Poissonian or sub-Poissonian dynamics for the neurons, whenever $I_{a}^{\left(\alpha \right)}$ is sub-threshold or supra-threshold (Lerchner et al., 2006).

**Figure 3:**
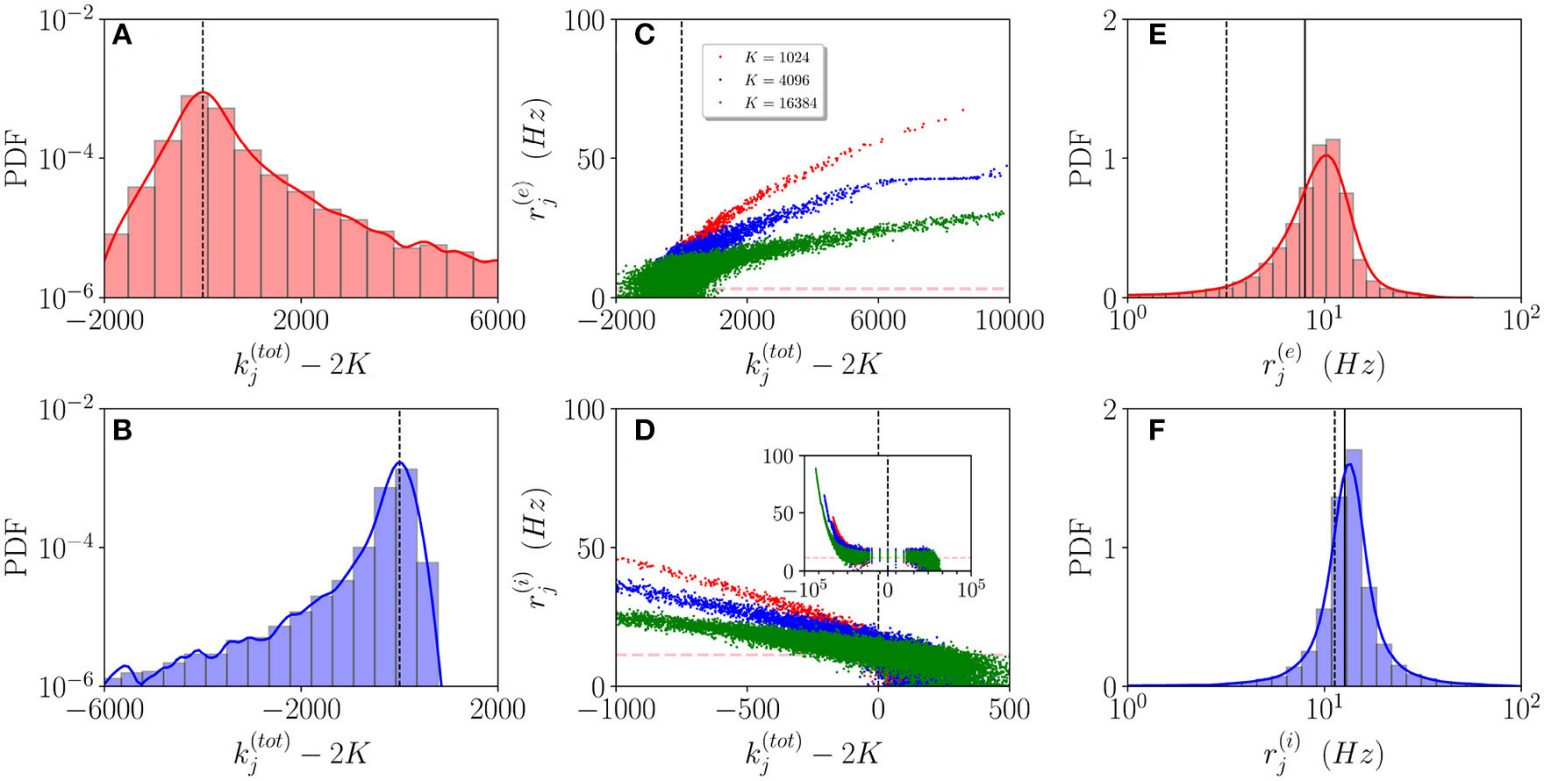
Asynchronous dynamics: Probability distribution functions (PDFs) of the total in-degrees $k_{j}^{\left(tot\right)}$ for excitatory **(A)** and inhibitory **(B)** active neurons for *K* = 16, 384. **(C,D)** Firing rates of the excitatory (inhibitory) neurons $r_{j}^{\left(e\right)}$ ($r_{j}^{\left(i\right)}$) vs. their total in-degrees $k_{j}^{\left(tot\right)}-2K$ symbols refer to *K* = 1,024 (red), *K* = 4,096 (blue), and *K* = 16,384 (green). The inset in **(D)** is an enlargement of the panel displaying the firing rates over the entire scale $k_{j}^{\left(tot\right)}-2K$. The magenta dashed lines in **(C,D)** represent the balanced state solution $\left({\bar{R}}_{0}^{\left(e\right)},{\bar{R}}_{0}^{\left(i\right)}\right)$. **(E,F)** PDF of the excitatory (inhibitory) firing rates $r_{j}^{\left(e\right)}$ ($r_{j}^{\left(i\right)}$) for *K* = 16, 384, the solid (dashed) line refers to the MF results ${\bar{R}}^{\left(x\right)}$ (${\bar{R}}_{0}^{\left(x\right)}$) with *x* = *e, i*. The red (blue) solid line refers to a log-normal fit to the excitatory (inhibitory) PDF with mean 8.8 Hz (17.5 Hz) and SD of 3.8 Hz (2.3 Hz). The parameters are the same as in Figure 1, the firing rates have been estimated by simulating the networks for a total time *T*_*s*_ = 60 s, after discarding a transient *T*_*t*_ = 40 s.

In order to understand how the in-degree heterogeneity influences the network dynamics at a microscopic level, we examine the dynamics of active neurons in the function of their total in-degree $k_{j}^{\left(tot\right)}$. This is defined for excitatory (inhibitory) neurons as $k_{j}^{\left(tot\right)}=k_{j}^{\left(ee\right)}+k_{j}^{\left(ei\right)}$ ($k_{j}^{\left(tot\right)}=k_{j}^{\left(ii\right)}+k_{j}^{\left(ie\right)}$). Furthermore, a neuron is considered as active if it has fired at least once during the whole simulation time *T*_*t*_ + *T*_*s*_ = 100 s, therefore, if it has a firing rate larger than 0.01 Hz. As shown in Figures 3A,B, the probability distribution function (PDF) of active neurons is skewed toward values $k_{j}^{\left(tot\right)}>2K$ ($k_{j}^{\left(tot\right)}<2K$) for excitatory (inhibitory) neurons. These results reflect the fact that the excitatory (inhibitory) neurons with low (high) recurrent in-degrees $k_{j}^{\left(ee\right)}<<K$ ($k_{j}^{\left(ii\right)}>>K$) are driven below the threshold by the inhibitory activity, that is predominant in the network since *R*^(*i*)^ > *R*^(*e*)^, $g_{0}^{\left(ei\right)}>g_{0}^{\left(ee\right)}$, and $g_{0}^{\left(ii\right)}>g_{0}^{\left(ie\right)}$. The number of silent neurons for *K* > 1,024 is of the order of 6-10% for both inhibitory and excitatory populations, in agreement with experimental results for the barrel cortex of mice (O'Connor et al., 2010), where a fraction of 10% of neurons was identified as silent with a firing rate slower than 0.0083 Hz. It should be remarked that all the population averages we report include the silent neurons.

Let us now examine how the firing rates of active neurons will modify by increasing the value of the median in-degree *K*. The single neuron firing rates as a function of their total in-degrees $k_{j}^{\left(tot\right)}$ are reported in Figures 3C,D for *K*=1,024, 4,096 and 16,384. A common characteristic is that the bulk neurons, those with $k_{j}^{\left(tot\right)}≃2K$, tend to approach the firing rate values $\left({\bar{R}}_{0}^{\left(e\right)},{\bar{R}}_{0}^{\left(i\right)}\right)$ (magenta dashed lines) corresponding to the expected solutions for a balanced network in the limit *N* >> *K* → ∞ (van Vreeswijk, 1996). This is confirmed by the analysis of their coefficient of variations *cv*_*j*_, whose values are of order one, as expected for fluctuation driven dynamics. On the other hand, the outlier neurons, i.e., those with $k_{j}^{\left(tot\right)}$ far from 2*K*, are all characterized by low values of the coefficient of variation *cv*_*j*_ indicating a mean driven dynamics. However, there is a striking difference between excitatory and inhibitory neurons. For the excitatory ones, we observe that the firing rates of the outliers with $k_{j}^{\left(tot\right)}>>2K$ decrease for increasing *K*, while for the inhibitory population the increase of *K* leads to the emergence of outliers at $k_{j}^{\left(tot\right)}<<2K$ with higher and higher firing rates (refer to the inset in Figure 3D). This difference can be explained by the different values measured for $I_{eff}^{\left(e\right)}$ and $I_{eff}^{\left(i\right)}$ in the network (refer to Figure 2C). The increase of *K* leads for the excitatory (inhibitory) population to the emergence of neurons with very large $k_{j}^{\left(ee\right)}>>K$ (very small $k_{j}^{\left(ii\right)}<<K$) whose dynamics should be supra-threshold. However, this is compensated in the excitatory case by the rapid drop of $I_{eff}^{\left(e\right)}$ toward zero or negative values, while for the inhibitory population $I_{eff}^{\left(i\right)}$ remains positive even at the largest *K* we have examined.

These outliers seem to have a negligible influence on the population dynamics, as suggested by the fact that the mean firing rates are reasonably well approximated by the balanced solutions ${\bar{R}}_{0}^{\left(e\right)}$ and ${\bar{R}}_{0}^{\left(i\right)}$ and as also confirmed by examining the PDFs of the firing rates for *K* = 16,384. As shown in Figures 3E,F, the excitatory (inhibitory) PDF can be well fitted by a log-normal distribution with a mean 8.8 Hz (17.5 Hz) and SD of 3.8 Hz (2.3 Hz). This is considered a clear indication that the network dynamics is fluctuation driven (Roxin et al., 2011) as confirmed by recent investigations in the hippocampus and the cortex (Wohrer et al., 2013; Buzsáki and Mizuseki, 2014; Mongillo et al., 2018), as well as in the spinal motor networks (Petersen and Berg, 2016). However, the relative widths of our distributions are narrower than those reported in Mongillo et al. (2018). This difference can find an explanation in the theoretical analysis reported in Roxin et al. (2011), where the authors have shown that quite counter intuitively a wider distribution of the synaptic heterogeneities can lead to a narrower distribution of the firing rates. Indeed, in this study, we consider Lorentzian distributed in-degrees, while in Mongillo et al. (2018) Erdös-Renyi networks have been analyzed. As a further aspect, we have estimated the number of inhibitory neurons firing faster than a certain threshold ν_*th*_, this number does not depend on the median in-degree for sufficiently large *K* > 5,000, however, it grows proportionally to *N*. In the considered cases, the fraction of these neurons is ≃1% for ν_*th*_ = 50 Hz.

From this analysis, we can conclude that at any finite *K* and for finite observation times, we have at a macroscopic scale an essentially balanced regime sustained by the bulk of active neurons, whose dynamics are fluctuation-driven. Furthermore, we also have a large body of silent neurons and a small fraction of mean driven outliers. These should be considered as typical features of finite heterogeneous neural circuits as shown in various experiments (O'Connor et al., 2010; Landau et al., 2016). Moreover, in the present case, we report quite different behaviors for outliers whose macroscopic effective input currents are supra- or sub-threshold.

### 3.3. Collective Oscillations

We will now characterize the different types of COs observable by first following a route to coherent chaos for the E-I balanced network and successively we will examine how oscillations exhibiting an abnormal level of synchronization, somehow similar to those observable during an ictal state in the brain (Lehnertz et al., 2009), can emerge in our system. Furthermore, we will consider the phenomenon of quasi-periodicity and frequency locking occurring for fluctuation driven oscillations. As the last issue, the scaling of the frequencies and amplitudes of COs with the in-degree and as a function of the external DC current is reported.

#### 3.3.1. A Period Doubling Route to Coherent Chaos

**Figure 4:**
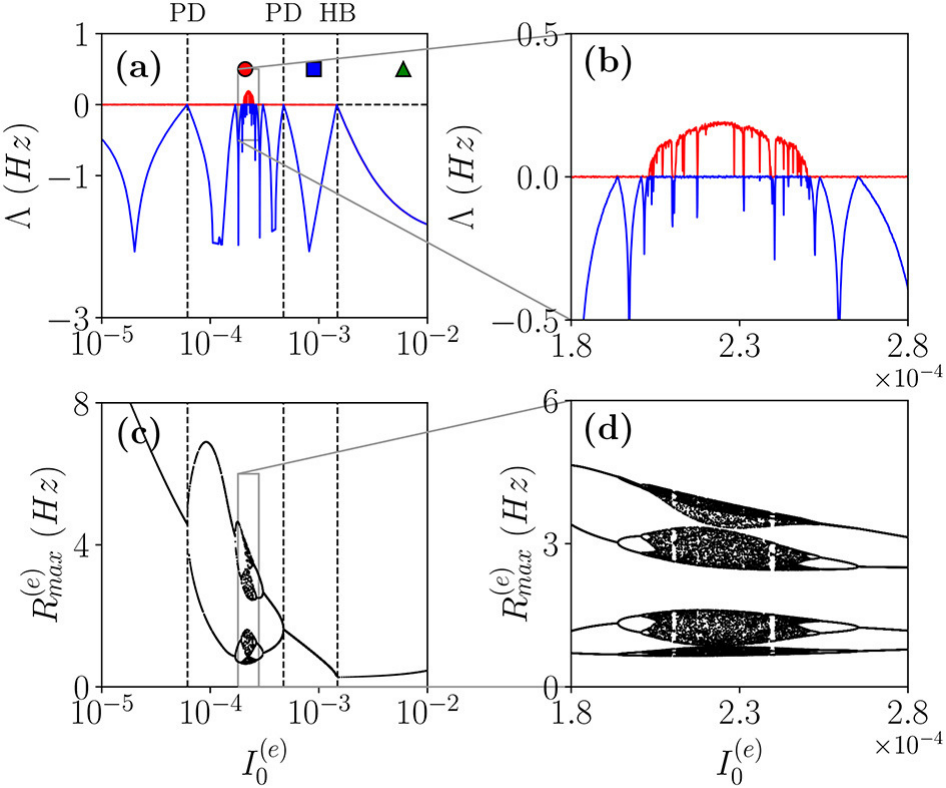
Coherent chaos. **(a,b)** First (red) λ_1_ and second λ_2_ (blue) (LEs) for the MF vs. the DC current $I_{0}^{\left(e\right)}$ for the parameter cut corresponding to the dashed black line in Figure 1A. The dashed vertical lines in **(a)** indicate a super-critical Hopf bifurcation (HB) from a stable focus to periodic COs and the region of the period doubling (PD) cascade. The symbols denote three different types of MF solutions: namely, stable focus (green triangle); periodic oscillations (blue square) and chaotic oscillations (red circle). **(c,d)** Bifurcation diagrams for the same region obtained by reporting the maximal value of the instantaneous firing rate *R*^(*e*)^ measured from MF simulations. The parameters are the same as in Figure 1, other parameters set as $\Delta_{0}^{\left(ii\right)}=0.3$, $\Delta_{0}^{\left(ee\right)}=2.0$, *K* = 1,000.

As a first case, we will follow the path in the parameter space denoted as a dashed black line in Figure 1A. In particular, in order to characterize the different dynamical regimes, we have estimated the LS {λ_*i*_} associated the MF equations. As shown in Figure 4, this analysis has allowed us to identify a period doubling cascade toward a chaotic region, characterized by periodic and chaotic windows. In particular, we observe a focus region (II) for $0.0015<I_{0}^{\left(e\right)}<50.6105$, the focus loses stability *via* a super-critical HB at $I_{0}^{e}≃0.0015$ giving rise to COs. One observes a period doubling cascade [regime (V)] taking place in the interval $I_{0}^{\left(e\right)}\in \left[0.00006177;0.00047297\right]$ followed by a regime of COs at lower values of $I_{0}^{\left(e\right)}$. The chaotic dynamics refer to the MF evolution, and it can be, therefore, definitely identified as collective chaos (Nakagawa and Kuramoto, 1993; Shibata and Kaneko, 1998; Olmi et al., 2011). A peculiar aspect of this period doubling cascade is that the chaotic dynamics remain always confined in four distinct regions without merging in a unique interval as it happens e.g., for the logistic map at the Ulam point (Ott, 2002). This is due to the fact that the population dynamics display period four oscillations characterized by four successive bursts, whose amplitudes (measured by $R_{max}^{\left(e\right)}$) varies chaotically but each one remains restricted in an interval not overlapping with the other ones.

**Figure 5:**
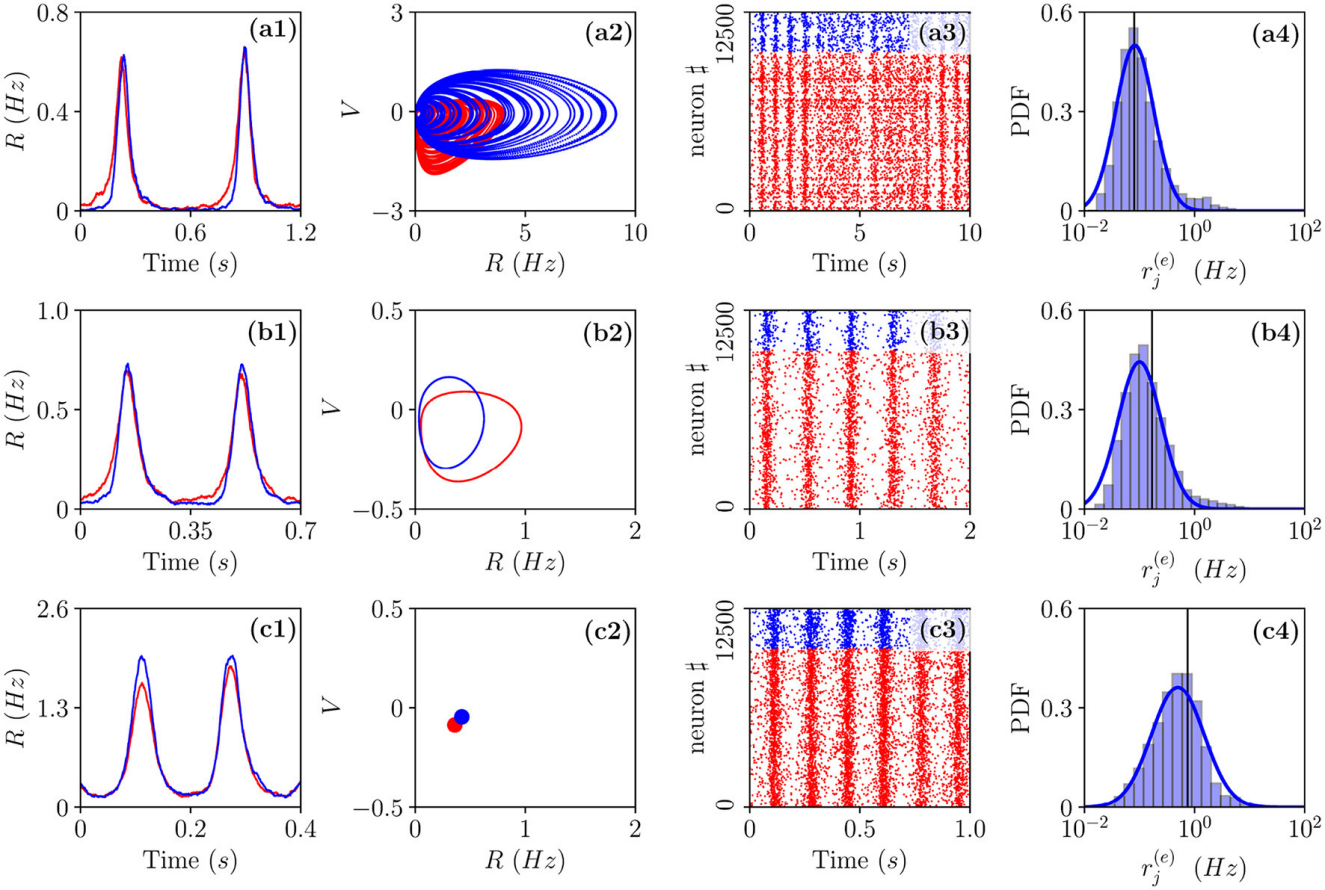
Different types of collective oscillations(COs). Row **(a)** refers to the chaotic state observable for $I_{0}^{\left(e\right)}=0.00021$ in the MF denoted by a red circle in Figure 4a; row **(b)** to the oscillatory state of the MF observable for $I_{0}^{\left(e\right)}=0.0009$ denoted by a blue square in Figure 4a; row **(c)** to the stable focus for the MF observable for $I_{0}^{\left(e\right)}=0.006$ denoted by a green triangle in Figure 4a. The first column displays the population firing rates vs. time obtained from the network dynamics, the second, the corresponding MF attractors in the planes identified by (*R*^(*e*)^, *V*^(*e*)^) and (*R*^(*i*)^, *V*^(*i*)^), the third, the raster plots, and the fourth, the PDFs of the excitatory firing rates $r_{j}^{\left(e\right)}$. Red (blue) color refers to excitatory (inhibitory) populations, the solid vertical lines in column 4 to the mean firing rate and the blue solid line to a fit to a log-normal distribution. Parameters as in Figure 2, apart from $\Delta_{0}^{\left(ii\right)}=0.3$, $\Delta_{0}^{\left(ee\right)}=2.0$, *K* = 1,000. For the estimation of the firing rates we employed *N*^(*e*)^ = 40,000 and *N*^(*i*)^ = 10,000, while for the raster plots, *N*^(*e*)^ = 10,000 and *N*^(*i*)^ = 2,500. The total integration time has been of 120 s after discarding a transient of 80 s.

Let us now examine the network dynamics for the three peculiar MF solutions indicated in Figure 4a corresponding to a stable focus (II) characterized by LE (λ_1_ = λ_2_ = −0.0299, λ_3_ = λ_4_ = −0.101) for $I_{0}^{\left(e\right)}=0.006$ (green triangle), to a stable oscillation (IV) with (λ_1_ = 0.0, λ_2_ = −0.0343, λ_3_ = −0.0555, λ_4_ = −0.1732) for $I_{0}^{\left(e\right)}=0.0009$ (blue square), and to collective chaos (v) with (λ_1_ = 0.0033, λ_2_ = 0.0, λ_3_ = −0.0809, λ_4_ = −0.1855) for $I_{0}^{\left(e\right)}=0.00021$ (red circle). As shown in Figure 5, for all these three cases, the network dynamics is always characterized by oscillations: namely, O_P_ for the regimes (IV) and (V) and fluctuation induced O_F_ for to the stable MF focus.

A typical feature of the O_P_ oscillations is that the excitatory neurons start to fire followed by the inhibitory ones, furthermore, the peak of activity of the excitatory population usually precedes that of the inhibitory neurons of a time interval Δ*t*. Then the inhibitory burst silences the excitatory population for the time needed to recover toward the firing threshold. This recovering time sets the frequency ν_*CO*_ of the COs. In our set-up, the excitatory bursts are wider than the inhibitory ones due to the fact that $\Delta_{0}^{\left(ee\right)}>\Delta_{0}^{\left(ii\right)}$. All these features are quite evident from the population firing rates shown in Figures 5a1,b1 and the raster plots in panels Figures 5a3,b3. These are typical characteristics of a PING-like mechanism reported for the generation of γ oscillations in the cortex (Tiesinga and Sejnowski, 2009), despite the fact that the frequencies of the COs shown in panels (a) and (d) are of the order of few Hz. Fluctuation driven oscillations O_F_ emerging in the network are radically different, as shown in Figure 5c1, in this case, the excitatory and inhibitory populations deliver almost simultaneous bursts. Further differences among O_P_ and O_F_ oscillations can be identified at the level of single neuron activity. These can be appreciated by considering the PDFs of the excitatory firing rates $r_{j}^{\left(e\right)}$ reported in the fourth column of Figure 5. As shown in Figure 5c4 these firing rates are log-normally distributed for O_F_ oscillations, thus, confirming their fluctuation driven origin (Roxin et al., 2011; Petersen and Berg, 2016). On the other hand, for O_P_ oscillations, we observe with respect to a log-normal distribution an excess of high firing neurons and a lack of low firing ones (refer to Figures 5a4,b4). This seems to indicate the presence of a larger number of mean driven excitatory neurons. Indeed this is the case, for $I_{0}^{\left(e\right)}=$ 0.00021 and $I_{0}^{\left(e\right)}=$ 0.0009, the percentage of active excitatory neurons driven by average effective currents supra-threshold $i_{eff,j}^{\left(e\right)}$ is ≃ 1.7 − 1.2%, while for $I_{0}^{\left(e\right)}=$0.006, it drops to ≃0.6%. The percentage of active inhibitory neurons on average supra-threshold is quite limited in both cases being of the order of 0.25–0.13%. Another interesting feature distinguishing the two kinds of oscillations is the fact that for O_P_, the excitatory supra-threshold neurons have a firing rate $r_{j}^{\left(e\right)}>\nu_{CO}$ and that the few neurons with firing rates locked to ν_*CO*_ are on average exactly balanced, i.e., they have $i_{eff,j}^{\left(e\right)}≃0$. The situation is different for the O_F_ oscillations, where we observe a group of sub-threshold excitatory and inhibitory neurons firing locked with the population bursts. In both cases, most parts of neurons are definitely sub-threshold firing at frequencies smaller than ν_*CO*_, as expected for an E-I balanced network displaying fast network oscillations associated with irregular neural discharges (Brunel and Wang, 2003).

**Figure 6:**
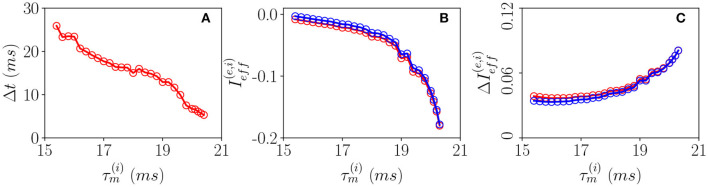
Pyramidal-interneuron gamma (PING)-like O_*P*_ COs. **(A)** Firing delays Δ*t* between the excitatory population peak and the inhibitory one vs. $\tau_{m}^{\left(i\right)}$. Effective mean input currents (Equation 13) **(B)** and current fluctuations (Equation 20) **(C)** vs. $\tau_{m}^{\left(i\right)}$, the excitatory (inhibitory) population are denoted by red (blue) circles. All the data reported in this study refer to MF simulations. The parameters are $I_{0}^{\left(e\right)}=0.0009$, $\Delta_{0}^{\left(ii\right)}=0.3$, $\Delta_{0}^{\left(ee\right)}=2.0$, *K* = 1,000, and $\tau_{m}^{\left(e\right)}=20$ ms.

In order to understand the different mechanisms at the basis of O_P_ and O_F_ oscillations, let us examine how the delay Δ*t* between excitatory and inhibitory bursts, observed for O_P_ oscillations, modifies as a function of the membrane time constant of the inhibitory population $\tau_{m}^{\left(i\right)}$. An increase of $\tau_{m}^{\left(i\right)}$ of ≃5 ms has the effect of reducing the delay of almost a factor six from Δ*t* ≃ 28 ms to Δ*t* ≃ 5 ms, as shown in Figure 6A. The increase of $\tau_{m}^{\left(i\right)}$ leads to an enhanced inhibitory action since the integration of the inhibitory membrane potentials occurs on longer time scales, and this promotes a higher activity of the inhibitory population. Indeed, this is confirmed from the drop of the effective input currents from an almost balanced situation where the average $I_{eff}^{\left(e\right)}$ and $I_{eff}^{\left(i\right)}$ are almost zero to a situation where they are definitely negative (refer to Figure 6B). Thus, for increasing $\tau_{m}^{\left(i\right)}$, the percentage of neurons below threshold also increases and as a consequence the dynamics become more and more noise driven, as testified by the increase of the current fluctuations $\Delta I_{eff}^{\left(e,i\right)}$ as shown in Figure 6C. In summary, the delay is due to the fact that despite the effective inhibitory and excitatory currents are essentially equal, as shown in Figure 6B, the wider distribution of the excitatory in-degrees promotes the presence of excitatory neurons supra-threshold that are the ones igniting the excitatory burst before the inhibitory one. The delay Δ*t* decreases whenever the number of these supra-threshold neurons decreases, and it will vanish when the dynamics will become essentially fluctuation driven as in the case of O_F_ oscillations.

#### 3.3.2. From Fluctuation Driven to Abnormally Synchronized Oscillations

**Figure 7:**
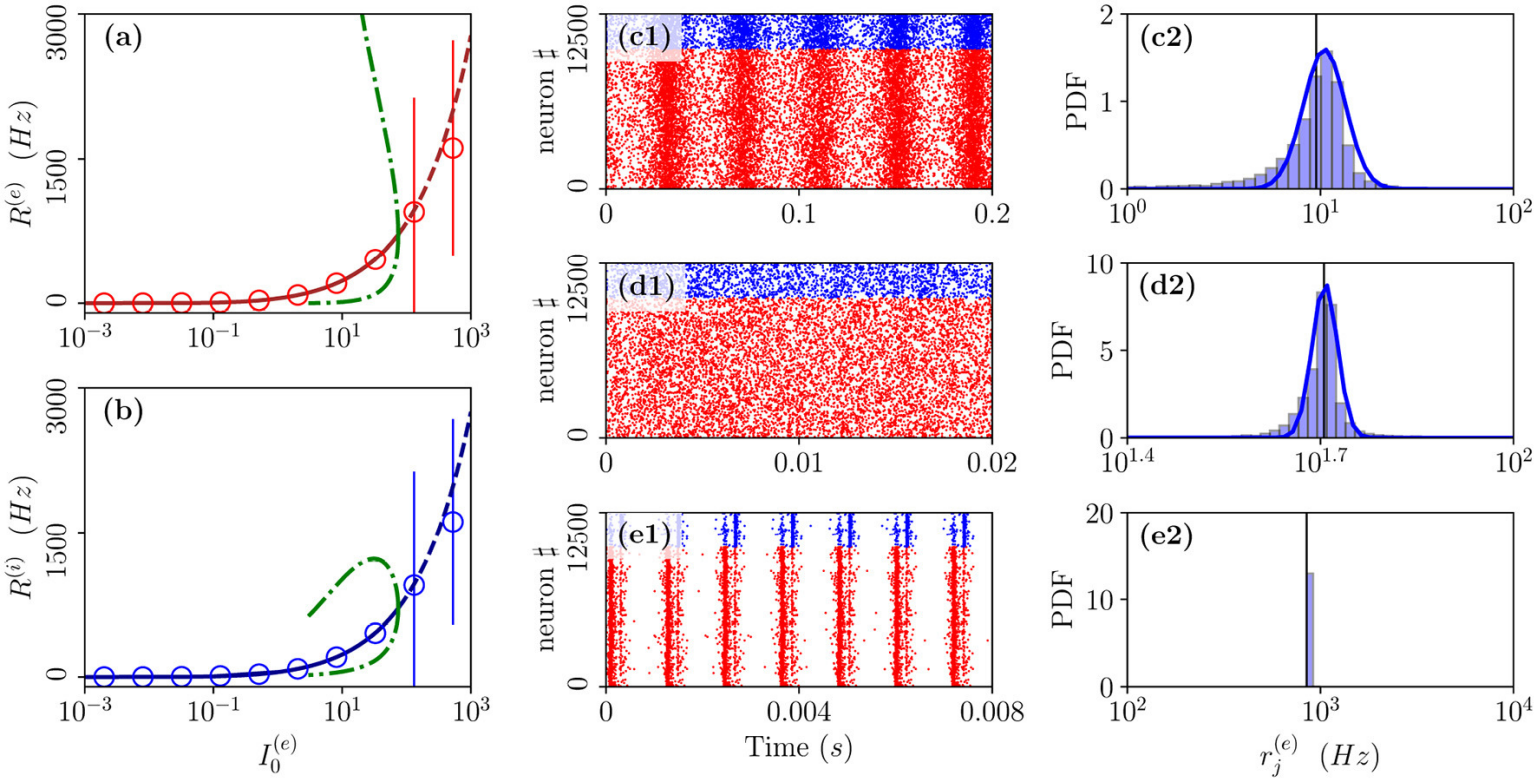
From fluctuation driven to abnormally synchronized oscillations. Firing rates *R*^(*e*)^ **(a)** and *R*^(*i*)^ **(b)** as a function of $I_{0}^{\left(e\right)}$ for E-I network (circles) and neural mass model (lines) for the parameter cut corresponding to the dashed purple line in Figure 1A. For the neural mass model: solid (dashed) line shows stable (unstable) focus solution ${\bar{R}}^{\left(e\right)}$ and ${\bar{R}}^{\left(i\right)}$; green dot-dashed lines refer to the extrema of *R*^(*e*)^(*R*^(*i*)^) for the unstable LC present in region (II). The unstable LC emerges at the sub-critical HB for $I_{0}^{\left(e\right)}=74.1709$ separating region (II) from (I), where the focus becomes unstable. Raster plots and PDFs of the excitatory firing rates $r_{j}^{\left(e\right)}$ are reported for specific cases: namely, $I_{0}^{\left(e\right)}=0.128$ **(c1,c2)**, $I_{0}^{\left(e\right)}=1.024$ **(d1,d2)**, and $I_{0}^{\left(e\right)}=100$ **(e1,e2)**. The solid vertical lines in **(c2,d2,e2)** refer to the mean firing rate. Parameters as in Figure 1, other parameters are set as $\Delta_{0}^{\left(ii\right)}=0.3$, $\Delta_{0}^{\left(ee\right)}=1.58$, *K* = 1,000 *N*^(*e*)^ = 10,000, and *N*^(*i*)^ = 2,500.

As the second range of parameters, we consider the cut in the parameter plane shown in Figure 1A as a purple dashed line. For these parameters, we report in Figures 7a,b the average in time of the excitatory and inhibitory population rate as a function of the excitatory DC current $I_{0}^{\left(e\right)}$. In particular, we compare network simulations (red and blue circles) with the MF results (red and blue lines). These predict a stable focus (solid lines) up to $I_{0}^{\left(e\right)}=74.1709$, where a sub-critical HB destabilizes such solution giving rise to an unstable focus (dashed lines). In panel (a), and (b), we have also reported as green dot-dashed lines the extrema of *R*^(*e*)^ and *R*^(*i*)^ corresponding to the unstable oscillations emerging at the HB. For currents below the HB, we observe a good agreement among the average network activity and the MF results.

In particular, below the HB, while the MF predicts only the existence of a stable focus, the network dynamics reveals quite interesting features. As shown in Figure 7d1, the system dynamics is indeed asynchronous for intermediate current values, in this study, $I_{0}^{\left(e\right)}=1.024$, however, at lower currents, we observe fluctuation driven oscillations O_F_ as evident from the raster plot displayed in Figure 7c1 for $I_{0}^{\left(e\right)}=0.128$. As shown in Figures 7c2,d2, both these regimes are characterized by log-normal distributions of the firing rates, thus, indicating that the dynamics are fluctuation driven.

As reported in Montbrió et al. (2015), when the network dynamics become strongly synchronous (as expected for very high excitatory DC external current), the MF formulation fails since the population rates predicted within the MF formulation diverge. However, as shown in Figures 7e1,e2, due to finite size effects, we observe in the network a strong synchronous COs of type O_S_ corresponding to the MF region (I) where the MF model predicts no stable solution. These abnormally synchronized oscillations are also characterized by a quite fast frequency of oscillation ν_CO_ ≃ 800 − 1,000 Hz. Furthermore, similarly to the O_P_ oscillations, they emerge due to a PING-like mechanism. This is evident from the raster plot in Figure 7e1, where excitatory neurons fire almost synchronously followed, after an extremely short delay, by the inhibitory ones whose activity silence all the network until the next excitatory burst. Quite astonishingly, the mean population rates measured in the network are reasonably well captured by the MF solutions associated with the unstable focus even beyond the HB, despite the network is now displaying COs (as shown in Figures 7a,b).

**Figure 8:**
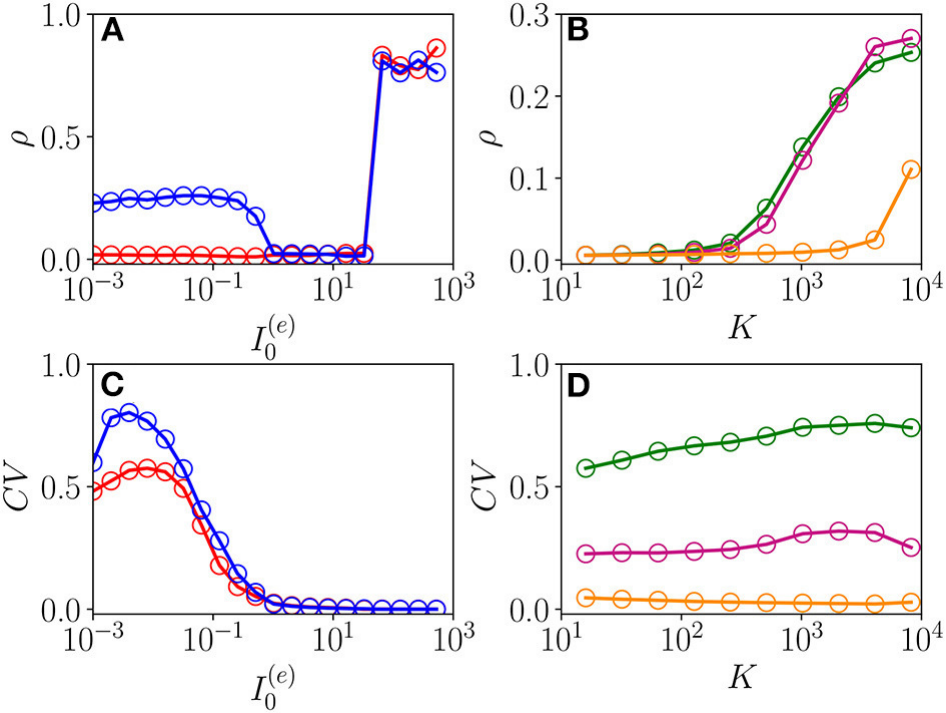
From fluctuation driven to abnormally synchronized oscillations. Coherence indicator ρ (Equation 4) for the whole network of excitatory and inhibitory neurons vs. the excitatory DC current $I_{0}^{\left(e\right)}$ **(A)** and the median in-degree *K* **(C)**. Coefficient of variation *CV* for the whole network vs. $I_{0}^{\left(e\right)}$ **(B)** and *K* **(D)**. In **(A,C)**, the symbols refer to different values of the median in-degree:namely, *K* = 100 (red circles) and *K* = 4,000 (blue circles). In **(B,D)**, the symbols refer to different excitatory DC currents: namely, $I_{0}^{\left(e\right)}=0.01$ (green circles), $I_{0}^{\left(e\right)}=0.1$ (purple circles), and $I_{0}^{\left(e\right)}=1.0$ (orange circles). Parameters as in Figure 1, other parameters $\Delta_{0}^{\left(ii\right)}=0.3$, $\Delta_{0}^{\left(ee\right)}=1.58$, *N*^(*e*)^ = 40,000, and *N*^(*i*)^ = 10,000.

The emergence of COs in the network can be characterized in terms of the coherence indicator ρ (Equation 4) for the whole population of neurons. This indicator is reported in Figure 8A as a function of $I_{0}^{\left(e\right)}$ for the same parameters previously discussed in Figure 7 and for two different values of the median in-degree : *K* = 100 (red circles) and *K* = 4,000 (blue circles). For both values of *K*, we observe an almost discontinuous transition in the value of the coherence indicator at the sub-critical HB from $\rho ≃1/\sqrt{N}$, expected for an asynchronous dynamics, to values ρ≃1 corresponding to full synchronization. This discontinuous transition leads to the emergence of abnormally synchronized oscillations O_S_ in the network. Moreover, at sufficiently high in-degrees, we observe the emergence of a new coherent state for low DC currents $I_{0}^{\left(e\right)}<$ 1.024 characterized by a finite value of the coherence indicator, namely, ρ≃0.3. The origin of these oscillations can be better understood by examining the coefficient of variation *CV* averaged over the whole population, this is reported in Figure 8C for the same interval of excitatory DC current and the same in-degrees as in Figure 8A. It is evident that the *CV* assumes finite values only for small input currents, namely $I_{0}^{\left(e\right)}<1.024$, indicating the presence of not negligible fluctuations in the network dynamics. Furthermore, by increasing *K*, these fluctuations, as measured by the *CV*, increase as expected for a balanced network. This analysis suggests that these oscillations cannot exist in absence of fluctuations in the network, and therefore, they are of the O_F_ type. Furthermore, the network should be sufficiently connected in order to sustain these COs, as one can understand from Figures 8B,D, where ρ and *CV* are reported as a function of *K* for three different values of $I_{0}^{\left(e\right)}$. Indeed, for these parameter values, no O_F_ oscillation is observable for *K* < 400, even in presence of finite values of the *CV*.

As previously discussed in di Volo and Torcini (2018), the balance between excitation and inhibition generates endogenous fluctuations that modify the collective dynamics with respect to that predicted by the MF model, where the heterogeneity of the input currents, due to distributed in-degrees, is taken in account only as a quenched form of disorder and not as a dynamical source of the noise. However, also from this simplified MF formulation, one can obtain relevant information on the O_F_ oscillations, indeed as we will see in the next sub-section, the relaxation frequencies toward the stable MF focus represent a good estimation of the oscillation frequencies measured in the network. This suggests that the fluctuations present at the network level can sustain COs by continuously exciting the focus observed in the effective MF model with quenched disorder.

#### 3.3.3. Fluctuation Driven Oscillations: From Quasi-Periodicity to Frequency Locking

**Figure 9:**
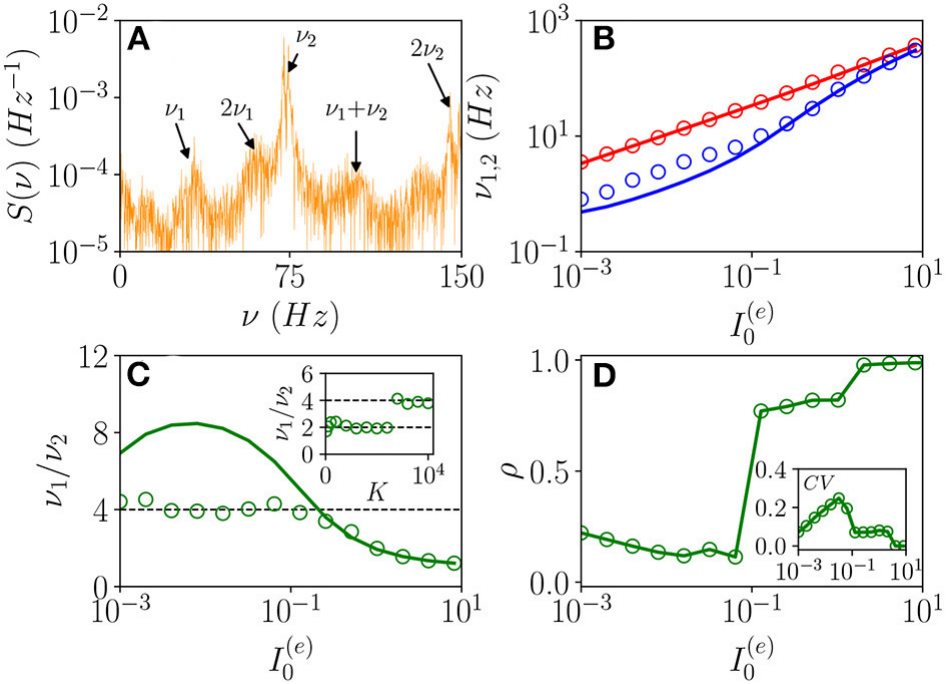
From quasi-periodicity to frequency locking. **(A)** Power spectra *S*(ν) of the mean membrane potential obtained from network simulations. **(B)** The two fundamental frequencies ν_1_(ν_2_) vs. $I_{0}^{\left(e\right)}$. **(C)** Frequency ratio ν_1_/ν_2_ vs. $I_{0}^{\left(e\right)}$, in the inset ν_1_/ν_2_ is shown vs. *K*. **(D)** Coherence parameter ρ vs. $I_{0}^{\left(e\right)}$, in the inset the corresponding *CV* is reported. In **(B,C)**, the symbols (solid lines) refer to ν_1_ and ν_2_ as obtained from the peaks of the power spectra *S*(ν) for *V*(*t*) obtained from the network dynamics (to the two relaxation frequencies $\nu_{1}^{R}$ and $\nu_{2}^{R}$ associated to the stable focus solution for the MF). Parameters as in Figure 1, other parameters are set as $\Delta_{0}^{\left(ii\right)}=0.3$, $\Delta_{0}^{\left(ee\right)}=1.58,N^{\left(e\right)}=$80,000, *N*^(*i*)^ = 20,000, *K* = 8,192, and $I_{0}^{\left(e\right)}=$ 0.128 in the inset of **(C)**.

As announced, this sub-section will be devoted to the characterization of the fluctuation driven oscillations O_*F*_ emerging in the region (II) reported in Figure 1. As the MF is now characterized by a stable focus with two couples of complex conjugate eigenvalues, there are two frequencies that can be excited by the irregular firing of neurons. Accordingly, as reported in di Volo and Torcini (2018), we expect the collective dynamics to be characterized by quasi-periodic dynamics with two (incommensurable) frequencies. These frequencies can be estimated by computing the power spectrum *S*(ν) of global quantities, e.g., the mean membrane potential *V*(*t*). In the case of periodic dynamics, *S*(ν) is characterized by one main peak in correspondence of the CO frequency and minor peaks at its harmonics, while in the quasi-periodic case, the power spectrum shows peaks located at the two fundamental frequencies and all their linear combinations. Indeed, as shown in Figure 9A, the power spectrum exhibit several peaks over a continuous profile and the peak frequencies can be obtained as a linear combination of two fundamental frequencies (ν_1_, ν_2_). As already mentioned, the noisy background is due to the fluctuations present in the balanced network. It is evident from Figure 9B, that these two fundamental frequencies are well reproduced by the two relaxation frequencies $\nu_{1}^{R}$ and $\nu_{2}^{R}$ toward the MF focus, in particular for $I_{0}^{\left(e\right)}\geq 0.256$. At smaller currents, while the first frequency is well reproduced by $\nu_{1}^{R}$, the second one is under-estimated by $\nu_{2}^{R}$. This is due to the phenomenon of frequency locking among the two collective rhythms present in the system: when the two frequencies become commensurable, we observe a common periodic CO. The locking order can be estimated by plotting the ratio between the two frequencies, indeed for low currents and *K* = 8, 192, the ratio is almost constant and equal to four denoting a 1:4 frequency locking (see Figure 9C). Furthermore, by fixing $I_{0}^{\left(e\right)}=0.128$ and by varying *K* the ratio ν_1_/ν_2_ can display different locked states, passing from locking of type 1:2 at low *K*, to 1:4 at larger values, as shown in the inset of Figure 9C.

As evident from Figures 9B,C, the locking phenomenon arises only in the network simulations and is not captured by the MF model. Furthermore, frequency locking occurs at low currents $I_{0}^{\left(e\right)}<0.1$ where the dynamics of the neurons are driven by the intrinsic current fluctuations present in the network but not in the MF. Indeed for low DC currents the level of synchronization within the populations measured by ρ decreases with $I_{0}^{\left(e\right)}$, while the *CV* increases (as shown in Figure 9D). These features suggest that this phenomenon is somehow similar to what was reported in Meng and Riecke (2018) for two coupled inhibitory neural populations subject to external uncorrelated noise. Meng and Riecke (2018) observed an increase of the locking region among collective rhythms by increasing the amplitude of the additive noise terms, this joined to a counter-intuitive decrease of the level of synchronization among the neurons within each population. However, in Meng and Riecke (2018), the neurons are subject to independent external noise sources, while in our case, the sources of fluctuations are intrinsic to the system and induced by the structural heterogeneity. Due to the network sparseness, the current fluctuations experienced by each neuron can be assumed to be indeed uncorrelated (Brunel and Hakim, 1999). Therefore, we are facing a new phenomenon that we can identify as a frequency locking of collective rhythms promoted by self-induced uncorrelated fluctuations. Indeed, the locking disappears for increasing external DC currents $I_{0}^{\left(e\right)}>0.1$, when the coherence parameter ρ displays an abrupt jump toward higher values and the *CV*≃0, thus, indicating that in this regime, the neuron dynamics becomes essentially mean driven and highly synchronized.

#### 3.3.4. Features of COs for Large In-degrees and DC Currents

The dynamics of balanced networks are usually characterized in the limit *N* >> *K* >> 1 by the emergence of a self-sustained asynchronous regime. However, LC solutions have been already reported for balanced networks in the seminal article van Vreeswijk and Sompolinsky (1996). These solutions can be either unbalanced or balanced, however, in this latter case, they were characterized by oscillations of vanishing small amplitude. van Vreeswijk and Sompolinsky (1996) have shown that balanced COs are not observable in their model in the limit *N* >> *K* → ∞ but only for finite *K*. Therefore, it is important to address in our case if COs can still be observable in the limit *N* >> *K* >> 1. Thus, in the following, we will investigate the dependence of COs features on the median in-degree *K* and the external DC currents.

**Figure 10:**
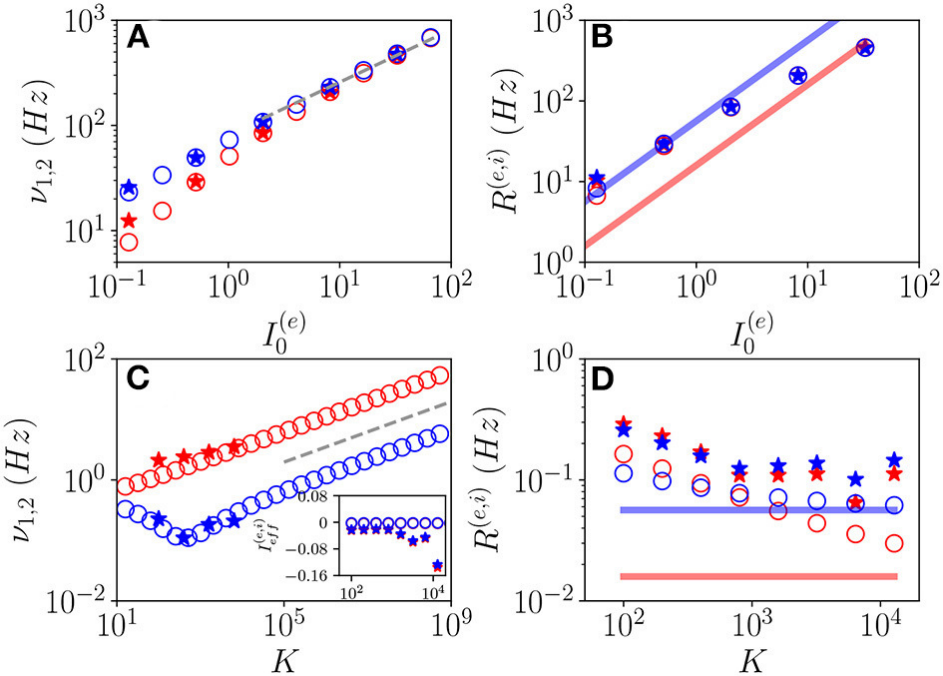
Frequencies and amplitudes of O_*F*_ oscillations. The two fundamental frequencies ν_1_ and ν_2_ vs. $I_{0}^{\left(e\right)}$ **(A)** and *K* **(C)** and the average firing rates vs. $I_{0}^{\left(e\right)}$ **(B)** and *K* **(D)** for the excitatory (red) and inhibitory (blue) populations. In the inset in **(C)**, the effective mean input currents $I_{eff}^{\left(e\right)}$ ($I_{eff}^{\left(i\right)}$) of the excitatory (inhibitory) population are shown vs. *K*. The dashed line in **(A,C)** corresponds to a power law-scaling $\propto {I_{0}^{\left(e\right)}}^{1/2}$ (∝*K*^1/4^) for the frequencies of the COs. The solid red (blue) line in **(B,D)** denotes the asymptotic MF result ${\bar{R}}^{\left(e\right)}$ (${\bar{R}}^{\left(i\right)}$). Network (MF) simulations are denoted as stars (circles). The MF data refer to the stable focus, in particular, in **(A,C)**, these are the two relaxation frequencies $\nu_{1}^{R}$ and $\nu_{2}^{R}$. Parameters as in Figure 1, other parameters: **(A,B)** *K* = 1, 000, $\Delta_{0}^{\left(ee\right)}=1.58$, $\Delta_{0}^{\left(ii\right)}=0.3$; **(C,D)** $I_{0}^{\left(e\right)}=0.001$, $\Delta_{0}^{\left(ee\right)}=1.3$, $\Delta_{0}^{\left(ii\right)}=0.3$; for the network simulations, we employed *N*^(*e*)^ = 80,000 and *N*^(*i*)^ = 20,000.

Let us first consider fluctuation driven O_*F*_ oscillations, in this case, we have an analytical prediction (Equation 19) for the scaling of the fundamental frequencies $\nu_{k}^{R}$ associated with the relaxation toward the macroscopic focus, which should grow proportionally to $\sqrt{I^{\left(e\right)}}$. As shown in Figures 10A,C, indeed this scaling is observable for sufficiently large *K* and $I_{0}^{\left(e\right)}$. It is also evident the extreme good agreement between results obtained from the network simulations and the theoretical predictions (Equation 19), at least for the values of *K* reachable with our simulations. Furthermore, the frequencies of COs cover an extremely large range of values from few Hz to KHz, and this range of frequencies can be spanned by varying either *K* or the external DC current $I_{0}^{\left(e\right)}$ as shown in Figures 10A,C.

To better characterize these regimes, we have also evaluated the average firing rates *R*^(*e*)^ and *R*^(*i*)^. These quantities are displayed for O_*F*_ oscillations in Figures 10B,D as a function of $I_{0}^{\left(e\right)}$ and *K*, respectively. From the network simulations (stars), we observe that *R*^(*e*)^ and *R*^(*i*)^ grow with $I_{0}^{\left(e\right)}$, and they are astonishingly quite well reproduced by the MF data (circles) for sufficiently large DC currents, despite the MF results refer to a stable focus and not to COs. Instead, at smaller currents (namely, $I_{0}^{\left(e\right)}=0.001$), the network data overestimates the MF results and the excitatory and inhibitory firing rates for *K* >> 1 seem to converge to a common constant value larger than those corresponding to the asynchronous regimes. For sufficiently large *K*, due to the prevalence of inhibition over excitation in the present model, we expect that the system will be sub-threshold, since the average excitatory and inhibitory firing rates are essentially coincident. Indeed this is confirmed by the analysis of the mean effective input currents $I_{eff}^{\left(e\right)}$ and $I_{eff}^{\left(i\right)}$ shown in the inset of Figure 10C. While for the MF focus, the dynamics appear as almost exactly balanced for all the considered median in-degree *K* since $I_{eff}^{\left(e\right)}≃I_{eff}^{\left(i\right)}≃0$, for the network dynamics $I_{eff}^{\left(e\right)}$ and $I_{eff}^{\left(i\right)}$ are definitely negative for *K* > 1, 000. This does not prevent the emergence of COs driven by fluctuations at large *K*, as indeed observed.

These results seem to indicate that for *N* >> *K* → ∞, the network will not converge in this case toward a balanced regime characterized by constant effective input currents. On the contrary from our analysis, it emerges that the system will become more and more sub-threshold for increasing *K* > 1,000. However, the system always exhibits fluctuation driven dynamics, since we measured *CV*≃ 0.6-0.8 at least in the range *K*≃100−10000 accessible to network simulations.

**Figure 11:**
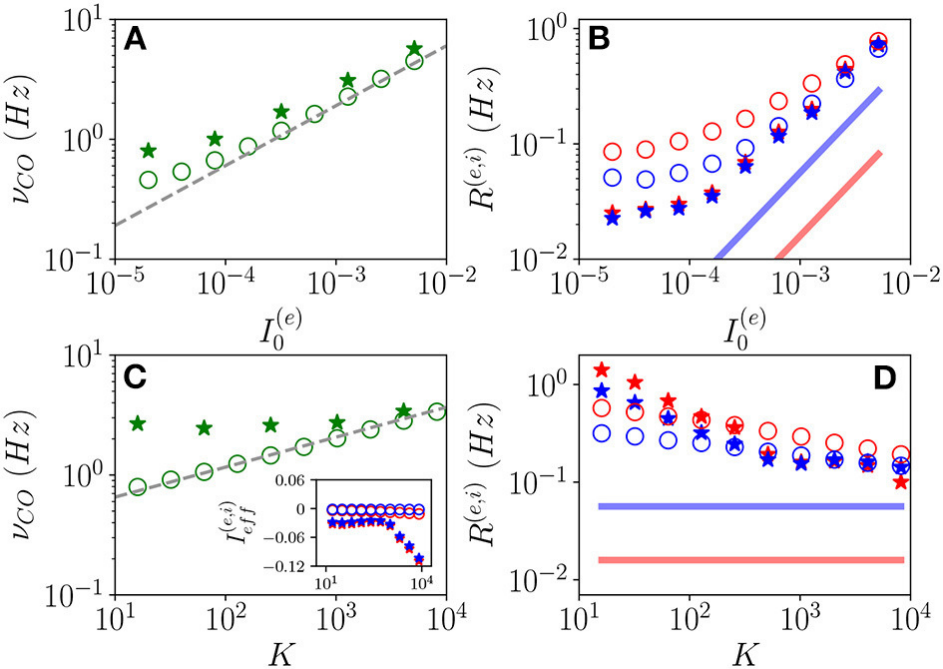
Frequencies and amplitudes of O_*P*_ oscillations. COs' frequency ν_*CO*_ vs. $I_{0}^{\left(e\right)}$ **(A)** and *K* **(C)** and mean firing rates vs. $I_{0}^{\left(e\right)}$ **(B)** and *K* **(D)** for the excitatory (red) and inhibitory (blue) populations. The dashed line in **(A,C)** corresponds to a power law-scaling $\propto {I_{0}^{\left(e\right)}}^{1/2}$ (∝*K*^1/4^) for the frequencies. In the inset in (c), the effective mean input currents $I_{eff}^{\left(e\right)}$ ($I_{eff}^{\left(i\right)}$) of the excitatory (inhibitory) population are shown vs. *K*. The solid red (blue) line in **(B,D)** denotes the asymptotic MF result ${\bar{R}}^{\left(e\right)}$ (${\bar{R}}^{\left(i\right)}$). The data obtained from network (MF) simulations are denoted as stars (circles). The data reported in **(A–D)** refer to the open circles in Figures 1A,B, respectively. For network simulations, we employed *N*^(*e*)^ = 80,000 and *N*^(*i*)^ = 20,000.

Let us now examine the O_*P*_ oscillations. As shown in Figures 11A,C, the frequencies ν_*CO*_ as estimated from the MF model (open circles) reveal an almost perfect increase proportional to $\sqrt{I^{\left(e\right)}}$ analogous to the one reported for O_*F*_ oscillations. The data obtained from network simulations (stars) converge toward the MF results for sufficiently large *K* and $I_{0}^{\left(e\right)}$.

The mean firing rates *R*^(*e*)^ and *R*^(*i*)^ grow with $I_{0}^{\left(e\right)}$ for fixed *K* and appear to converge toward a constant value for sufficiently large *K* for fixed $I_{0}^{\left(e\right)}$, refer to Figures 11B,D. Moreover, the network simulations (stars) approach the MF results (open circles) at large DC currents and median in-degrees. However, while in the MF the asymptotic values of *R*^(*e*)^ and *R*^(*i*)^ remain distinct even at large *K*, these seem to become identical in the network simulations. This reflects in the fact that while the MF is perfectly balanced in the whole range of examined in-degrees, since $I_{eff}^{\left(e\right)}≃I_{eff}^{\left(i\right)}≃0$, the network simulations reveal almost balanced effective input currents up to *K*≃ 1,000 and above such median in-degree a prevalence of the inhibitory drive (refer to inset of Figure 11C).

For both kinds of COs, we observe that while ν_*CO*_ diverges with *K*, the mean firing rates approach a constant value, thus, suggesting that the percentage of neurons participating in each population burst should vanish in the limit *K* → ∞. This result indicates that COs will finally disappear, however, more refined analyses are needed to derive the asymptotic behavior of the system in the large *K* limit, ref to di Volo et al. (2021) for a detailed discussion of this aspect for purely inhibitory networks.

## 4. Discussion

We have extensively characterized the macroscopic regimes emerging in a sparse balanced E-I network made of spiking QIF neurons with Lorentzian distributed in-degrees. The considered neuronal model joined to the peculiar choice of the distribution has allowed us to derive an exact low dimensional neural mass model describing the MF dynamics of the network in terms of the mean membrane potentials and of the population rates of the two populations (Montbrió et al., 2015; di Volo and Torcini, 2018). The low-dimensionality of the MF equations enabled us to study analytically the stationary solutions and their stability as well as to obtain the bifurcation diagrams associated with the model and to identify the possible macroscopic states.

### 4.1. Asynchronous Regimes

The stationary solutions of the MF correspond to the asynchronous regime, which is the regime usually analyzed in the context of balanced dynamics (van Vreeswijk and Sompolinsky, 1996; Renart et al., 2010; Litwin-Kumar and Doiron, 2012). In the present case, we have analytically obtained the stationary solutions for the mean membrane potentials and average firing rates for Lorentzian distributed in-degrees for any finite value of the median *K* and an HWHM scaling as $\Delta_{k}^{\left(\alpha \alpha \right)}=\Delta_{0}^{\left(\alpha \alpha \right)}{\left(K\right)}^{\eta }$ with η = 1/2. The MF estimations for the population firing rates are pretty well reproduced by the network simulations in the examined range of in-degrees *K*. Furthermore, from the analytic expression of the stationary firing rates (Equation 8), it is evident that for *K* > > 1, the asymptotic rates would not depend on the structural heterogeneity and correspond to those usually found for balanced homogeneous or Erdös-Renyi networks (van Vreeswijk and Sompolinsky, 1996; Monteforte and Wolf, 2010). This is due to the fact that the ratio ${\left(\Delta_{k}^{\left(\alpha \alpha \right)}\right)}^{2}/K$ remains constant for *K* → ∞. The final scenario will depend on the scaling exponent η, in particular, by assuming η = 3/4, the asymptotic firing rates ${\bar{R}}_{0}^{\left(\alpha \right)}$ will explicitly depend on the parameters $\Delta_{0}^{\left(\alpha \alpha \right)}$ controlling the structural heterogeneity. Whenever η > 3/4, the balanced state breaks down, and we face a situation similar to those investigated in Landau et al. (2016) and Pyle and Rosenbaum (2016)^1^.

However, despite the system approaching a balanced state, as testified by the fact that the effective input currents converge to finite values $I_{a}^{\left(\alpha \right)}$, and the current fluctuations stay finite for *K* → ∞, the balanced regime is not necessarily a sub-threshold one. Indeed, we have observed that we can have either sub-threshold or supra-threshold situations depending on the model parameters in agreement with the results previously reported in Lerchner et al. (2006). Moreover, the excitatory and inhibitory populations can achieve balanced regimes characterized by different asymptotic dynamics, where $I_{a}^{\left(i\right)}$ and $I_{a}^{\left(e\right)}$ have opposite signs.

While at a macroscopic level, the population activity for *N* >> *K* >> 1 approach is essentially that of a homogeneous balanced system, as shown in Figures 2A,B, the structural heterogeneity has a large influence on the single neuron dynamics, at least at finite *K* and finite investigation times. In particular, in analogy with experiments (Gentet et al., 2010; Mongillo et al., 2018), we considered a situation where the inhibitory drive prevails on the excitatory one. In this condition, microscopically the neural populations split into three groups: silent neurons, definitely sub-threshold; bulk neurons, which are fluctuation driven; and mean driven outlier neurons. In particular, excitatory (inhibitory) neurons with low (high) intra-population in-degrees are silenced due to the prevalence of synaptic inhibition. The silent neurons represent 6-10% of the whole population in agreement with experimental results for the mice cortex (O'Connor et al., 2010). Bulk neurons have in-degrees in the proximity of the median, and their firing rates approach the MF solution ${\bar{R}}_{0}^{\left(\alpha \right)}$ for increasing *K*. Outlier neurons represent a minority group almost disconnected from their own population, whose asymptotic behavior for *K* >> 1 is controlled by the sign of the effective mean input current.

### 4.2. Coherent Dynamics

The emergence of COs is observable in this balanced network whenever the level of heterogeneity in the inhibitory population is not too large, thus, suggesting that the coherence among inhibitory neurons is fundamental to support collective rhythms (Whittington et al., 2000). Indeed we observed two main mechanisms leading to COs: one that can be identified as PING-like and another one as fluctuation driven. The PING-like mechanism is present whenever the excitatory neurons are able to deliver an almost synchronous excitatory volley that in turn elicits a delayed inhibitory one. The period of the COs is determined by the recovery time of the excitatory neurons from the stimulus received from the inhibitory population. This mechanism is characterized by a delay between the firing of the pyramidal cells and the interneuronal burst as reported also in many experiments (Buzsáki and Wang, 2012). We have shown that this delay tends to vanish when the inhibitory action increases leading the system from a balanced situation to a definitely sub-threshold condition where the neural activity is completely controlled by fluctuations. In this latter case, the excitatory and inhibitory neurons fire almost simultaneously driven by the current fluctuations. These transform the relaxation dynamics toward a stable focus, observable in the MF, to sustained COs *via* a mechanism previously reported for inhibitory networks (di Volo and Torcini, 2018; Bi et al., 2020).

The PING-like COs undergo period doubling cascades by varying *K* and/or $I_{0}^{\left(e\right)}$ finally leading to collective chaos (Nakagawa and Kuramoto, 1993; Shibata and Kaneko, 1998). The nature of this chaotic behavior is definitely macroscopic since it is captured by the neural mass model obtained within the MF formulation, as shown by analyzing the corresponding LS. This kind of chaos implies irregular temporal fluctuations joined to coherence at the spatial level over a large part of the network resembling coherent fluctuations observed across spatial scales in the neocortex (Smith and Kohn, 2008; Volgushev et al., 2011; Okun et al., 2012; Achermann et al., 2016). Collective (or coherent) chaos has been previously shown to be a ubiquitous feature for balanced random spiking neural networks massively coupled, where *K* is proportional to *N* (Politi et al., 2018; Ullner et al., 2018). In this study, we have generalized such results to balanced random networks with sparse connectivity, where *K* is independent by *N*. Recently, it has been claimed that the presence of structured feed-forward connectivity in a random network is needed to observe coherent chaos (Landau and Sompolinsky, 2018). However, as evident from our results and those reported in Ullner et al. (2018) and Politi et al. (2018), coherent chaos can naturally emerge in a recurrent neural network in absence of any structured connectivity introduced *ad hoc* to promote collective behaviors. Furthermore, we have shown that collective chaos can emerge in random balanced networks with instantaneous synapses and the absence of any delay, refer to Ullner et al. (2018).

Fluctuation driven COs are usually observable in our system as quasi-periodic collective motions characterized by two incommensurate frequencies. However, whenever the current fluctuations become sufficiently strong, the two frequencies can lock and give rise to a collective periodic motion. Furthermore, the locking region is characterized by a low level of synchrony in the network. These results resemble those reported in Meng and Riecke (2018) for two interconnected inhibitory neural networks subject to external uncorrelated noise. In particular, the authors have shown that uncorrelated noise sources enhance synchronization and frequency locking among the COs displayed by the two networks, despite the reduced synchrony among neurons within each network. At variance with Meng and Riecke (2018), in our case, the noise sources are intrinsic to the neural dynamics, but they can be as well considered as uncorrelated due to the sparseness in the connections (Brunel and Hakim, 1999; Brunel, 2000). Therefore, we are reporting a new example of frequency locking among collective rhythms promoted by self-induced uncorrelated fluctuations.

According to analytical arguments, the frequencies of the COs grow proportionally to the square root of the excitatory DC current. This on one side allows simply by varying the parameters $I_{0}^{\left(e\right)}$ or *K*, to cover with our model a broad range of COs' frequencies analogous to those found experimentally in the cortex (Chen et al., 2017). On another side, it implies that the frequencies of COs diverge as *K*^1/4^, while the average firing rates seem to converge to a common value for sufficiently large *K*. These results seem to indicate that for large *K*, the network will become more and more unbalanced, with a prevalence of inhibition, while the amplitude of COs will tend to vanish. However, this analyses is not conclusive and more detailed analysis are required to capture the asymptotic behavior of the system in the limit *N* >> *K* >> 1.

### 4.3. Future Developments

The examined neural mass model has been derived by taking into account the random fluctuations due to the sparseness in the network connectivity only as a quenched disorder affecting the distribution of the effective synaptic couplings (Montbrió et al., 2015; di Volo and Torcini, 2018). The current fluctuations can be correctly incorporated in an MF formulation by developing a Fokker-Planck formalism for the problem, however, this will give rise to high (infinite) dimensional MF models (Brunel and Hakim, 1999; Brunel, 2000). We are currently developing reduction formalisms for the Fokker-Planck equation to obtain low dimensional neural mass models which will include the intrinsic current fluctuations (di Volo et al., 2021; Goldobin et al., 2021).

Relevant topics to investigate in the future to assess the generality of the reported results are their dependence on the chosen spiking neuron model and network architecture. In particular, for random networks, it is important to understand the role played by the distribution of the in-degrees, this is also in view of the recent findings reported in Klinshov et al. (2021).

## Data Availability Statement

The raw data supporting the conclusions of this article will be made available by the authors, without undue reservation.

## Author Contributions

HB and MV performed the simulations and data analysis. MV and AT were responsible for the state-of-the-art review and the article write-up. All the authors conceived and planned the research.

## Funding

AT received financial support by the Excellence Initiative I-Site Paris Seine (grant no. ANR-16-IDEX-008) (together with HB), by the Labex MME-DII (grant no. ANR-11-LBX-0023-01), by the ANR Project ERMUNDY (grant no. ANR-18-CE37-0014) (together with MV), and all part of the French programme Investissements d'Avenir.

## Conflict of Interest

The authors declare that the research was conducted in the absence of any commercial or financial relationships that could be construed as a potential conflict of interest.

## Publisher's Note

All claims expressed in this article are solely those of the authors and do not necessarily represent those of their affiliated organizations, or those of the publisher, the editors and the reviewers. Any product that may be evaluated in this article, or claim that may be made by its manufacturer, is not guaranteed or endorsed by the publisher.

## Footnotes

^1^In such cases, balance has been recovered either by rewiring the post-synaptic connections (Pyle and Rosenbaum, 2016) or by introducing some sort of homeostatic plasticity or of spike-frequency adaptation (Landau et al., 2016).
